# The role of tolvaptan add-on therapy in patients with acute heart failure: a systematic review and network meta-analysis

**DOI:** 10.3389/fcvm.2024.1367442

**Published:** 2024-05-30

**Authors:** Vireza Pratama, Jordan Budiono, Jarir At Thobari, Bambang Widyantoro, Vita Yanti Anggraeni, Lucia Kris Dinarti

**Affiliations:** ^1^ Faculty of Medicine Public Health and Nursing, Universitas Gadjah Mada, Yogyakarta, Indonesia; ^2^ Department of Cardiology, Gatot Soebroto Central Army Hospital (RSPAD), Jakarta, Indonesia; ^3^ Department of Cardiology and Vascular Medicine, Faculty of Medicine and Health Science, Universitas Pertahanan Republik Indonesia, Bogor, Indonesia; ^4^ Faculty of Medicine and Health Sciences, Atma Jaya Catholic University of Indonesia, Jakarta, Indonesia; ^5^ Department of Pharmacology and Therapy, Faculty of Medicine, Public Health and Nursing, Universitas Gadjah Mada, Yogyakarta, Indonesia; ^6^ Clinical Epidemiology and Biostatistic Unit (CEBU), Faculty of Medicine, Public Health and Nursing, Universitas Gadjah Mada, Yogyakarta, Indonesia; ^7^ Department of Cardiology and Vascular Medicine, Faculty of Medicine, Universitas Indonesia, National Cardiovascular Center Harapan Kita, Jakarta, Indonesia; ^8^ Department of Internal Medicine, Division of Cardiology, Faculty of Medicine, Public Health and Nursing, Universitas Gadjah Mada, Dr. Sardjito Hospital, Yogyakarta, Indonesia; ^9^ Department of Cardiology and Vascular Medicine, Faculty of Medicine, Public Health and Nursing, Universitas Gadjah Mada, Dr. Sardjito Hospital, Yogyakarta, Indonesia

**Keywords:** heart failure, acute heart failure, tolvaptan, network meta-analysis, systematic review

## Abstract

**Background:**

Several conflicting reviews have concluded that the use of loop diuretics is associated with poorer clinical and safety outcomes. Therefore, this study aimed to investigate the efficacy and safety of tolvaptan as an adjunct to conventional diuretic therapy in patients with acute heart failure (AHF).

**Methods:**

A comprehensive search was conducted on PubMed, Embase, ProQuest, EBSCO, and Cochrane Library until 24 May 2023 to identify randomized controlled trials that compared the effects of tolvaptan with conventional therapy and placebo in patients with AHF. The quality assessment of the included trials was conducted using the Cochrane risk of bias. A network meta-analysis (NMA) was conducted to examine the dosage effect of tolvaptan.

**Result:**

A total of 17 studies with 18 reports, involving 10,039 patients, were selected. The tolvaptan add-on therapy significantly alleviated dyspnea [24 h: RR 1.16 (1.04, 1.29), 48 h: RR 1.18 (1.04, 1.33)], reduced body weight within 48 h [Asian group, MD −0.93 (−1.48, −0.38); non-Asian group, MD −2.76 (−2.88, −2.65)], reduced edema [RR 1.08 (1.02, 1.15)], increased serum sodium [non-Asian group, MD 3.40 (3.02, 3.78)], and resulted in a change in serum creatinine [MD −0.10 (−0.18, −0.01)]. No significant differences were observed in mortality and rehospitalization. The NMA suggested that an intermediate dosage (15 mg/day) might offer the best efficacy in reducing dyspnea within 24 h, reducing edema, increasing serum sodium, and lowering the incidence of worsening renal function (WRF).

**Conclusion:**

In conclusion, the meta-analysis showed that tolvaptan contributed to the short-term alleviation of congestive symptoms, elevated sodium levels, and a lower incidence of WRF. However, no significant benefits were observed in long-term symptoms, rehospitalization rates, and mortality. An intermediate dosage of tolvaptan might be considered the optimal choice for various clinical outcomes.

**Systematic Review Registration:**

https://www.crd.york.ac.uk/, PROSPERO (CRD42023420288).

## 1 Introduction

Acute heart failure (AHF) is a commonly encountered condition that often leads to hospitalization. The wet-warm phenotype, which is characterized by congestive symptoms, is predominant in most AHF patients. Managing congestion through decongestive therapy is a primary focus in patient care (1). Historically, international guidelines have positioned furosemide, a loop diuretic, as the first-line medication for AHF. Despite its recognized benefits, furosemide comes with potential side effects, particularly the activation of the renin–angiotensin–aldosterone (RAA) system, which can have adverse effects on kidney function. This is particularly concerning in patients with heart failure (HF) with ejection fraction, where the RAA system may become overstimulated, thereby posing a potential risk in the presence of comorbid renal function disorders (2). Tolvaptan has been extensively studied for its decongestive effects without worsening renal function (WRF) (3), which formed the basis for conducting this study.

A current meta-analysis conducted by Kansara et al. (4) evaluated the short- and long-term effectiveness of tolvaptan in patients with AHF with standard HF care. The results showed that HF patients in the tolvaptan group experienced superior dyspnea relief, as shown by the Likert score, and a significant mean reduction in weight within the first 48 h (short term). However, it is crucial to acknowledge the potential impact of varying tolvaptan dosages used across the included studies, which could have influenced the results. To address this concern, we conducted an updated systematic review and a meta-analysis of randomized double-blinded tests, comparing tolvaptan add-on therapy with conventional diuretic therapy in hospitalized AHF patients. Our approach used a comprehensive network meta-analysis (NMA) to evaluate the dosage effect of tolvaptan. This meta-analysis aimed to provide insights into the efficacy and safety of tolvaptan add-on therapy in AHF patients, addressing the key questions related to its use in clinical practice.

## 2 Material and methods

### 2.1 Literature search

The search strategy adhered to the Preferred Reporting Items for Systematic Reviews and Meta-analyses (PRISMA) extension statement for NMA. The protocol was registered in the International Prospective Register of Systematic Reviews (PROSPERO) with identification number CRD42023420288. A systematic search was conducted on PubMed, Embase, EBSCO, ProQuest, and the Cochrane Library from inception to 24 May 2023. The applied keywords were “acute heart failure” OR “heart failure” AND “tolvaptan” OR “samsca” OR “opc 41061.” The general searching strategy was adjusted to the format in each database, as presented in detail in Supplementary S1. Additionally, the participant, index test, comparison, and outcome (PICO) detailed for this study are provided in Supplementary S2.

### 2.2 Study selection

The inclusion criteria for meta-analysis consisted of randomized trials of hospitalized patients with AHF, wherein tolvaptan served as an add-on to the conventional diuretic therapy in the therapy group, and comparisons were made with the therapy with placebo. The outcomes considered were dyspnea relief, changes in body weight, edema, electrolytes, mortality, and rehospitalization. The excluded outcomes were review articles, medical reports, observational studies, case reports, and editorials. Two independent reviewers (VP and JB) blindly evaluated the search results based on the inclusion and exclusion criteria. The third reviewer (BW) was consulted to make a decision when a consensus was not reached between VP and JB.

### 2.3 Outcomes

For each eligible study, efficacy indicators, including dyspnea relief, changes in body weight, edema reduction, changes in sodium level, and changes in creatinine level, were evaluated. Safety data, such as mortality and rehospitalization, were also analyzed. A subgroup analysis with NMA was conducted based on the different tolvaptan dosages (i.e., high, intermediate, and low).

### 2.4 Data extraction and quality assessment

The characteristics of the RCTs, consisting of the first reviewer, publication year, study acronym, study country, sample size, study period, intervention type(s), and clinical outcome(s), were summarized in Table 1. To investigate the potential biases in the analyzed RCTs, the Cochrane risk of bias assessment (Supplementary S3) was used (21). This means facilitating the evaluation of bias related to the random sequence era, allocation concealment, blinding of parties and analysts, blinding of the outcome reviews, particular reporting, incomplete outcome data, and other relevant metrics. Funnel plot asymmetry and Egger tests were applied to assess the potential evidence of publication bias (Supplementary S4).

**Table 1:** Basic characteristics of the included randomized clinical trials.

Author	Year	Acronym	Study location	Sample size	Study population	Follow-up	Intervention		Primary endpoints
							Tolvaptan	Control	
Lin et al. (5)	2020	NR	Taiwan	91	AHF	1–4 days and 1 month	Con + 15 mg/day of tolvaptan (*n* = 46)	Con + Placebo (*n* = 45)	[B], [C], [F]
Chitthai et al. (6)	2021	NR	Thailand	40	AHF	3 days	Con + 7.5 mg/day of tolvaptan (*n* = 20)	Con + Placebo (*n* = 20)	[D], [F], [G]
Tien Ng et al. (7)	2020	AQUA-HF	USA	33	AHF	4 days	Con + 30 mg/d of tolvaptan (*n* = 18)	Con + Placebo (*n* = 15)	[G}
Inomata et al. (8)	2018	K-STAR	Japan	81	AHF	7 days	Con + 15 mg/day of tolvaptan (*n* = 40)	Con + Placebo (*n* = 41)	[D], [E], [G]
Konstam et al. (9)	2017	SECRET of CHF	USA	240	AHF	7 days	Con + 30 mg/day of tolvaptan (*n* = 117)	Con + Placebo (*n* = 123)	[A], [B], [H]
Felker et al. (10)	2017	TACTISCS-HF	USA	257	AHF	48 h	Con + 30 mg/day of tolvaptan (*n* = 129)	Con + Placebo (*n* = 128)	[A], [B], [C], [F], [G], [E]
Tamaki et al. (11)	2017	NR	Japan	50	AHF	48 h	Con + 15 mg/day of tolvaptan (*n* = 26)	Con + Placebo (*n* = 24)	[G]
Jujo et al. (12)	2016	NR	Japan	60	AHF	5 days	Con + 7.5 mg/day of tolvaptan (*n* = 30)	Con + Placebo (*n* = 30)	[G], [H]
Shanmugam et al. (13)	2016	NR	India	51	AHF	5 days	Con + 15 mg/day of tolvaptan (*n* = 25)	Con + Placebo (*n* = 26)	[F], [H]
Kimura et al. (14)	2016	TACT-ADHF	Japan	52	AHF	7 days	Con + 7.5 mg/day of tolvaptan (*n* = 26)	Con + Placebo (*n* = 26)	[F]
Matsue et al. (3)	2016	AQUAMARINE	Japan	217	AHF	2 days	Con + 15 mg/day of tolvaptan (*n* = 108)	Con + Placebo (*n* = 109)	[A], [C], [E], [H]
Matsuzaki et al. (15)	2011	NR	Japan	110	AHF	7 days	Con + 30 mg/day of tolvaptan (*n* = 53)	Con + Placebo (*n* = 57)	[D]
Udelson et al. (16)	2011	NR	USA	42	AHF	8 days	Con + 30 mg/day of tolvaptan (*n* = 20)	Con + Placebo (*n* = 22)	[D], [F]
Udelson et al. (17)	2007	NR	USA	240	AHF	8 days	Con + 30 mg/day of tolvaptan (*n* = 120)	Con + Placebo (*n* = 120)	[H], [I]
Gheorghiade et al. (18) (Trial A)	2007	EVEREST	20 countries^a^	2,048	AHF	7 days	Con + 30 mg/day of tolvaptan (*n* = 1,018)	Con + Placebo (*n* = 1,030)	[A]
Gheorghiade et al. (18) (Trial B)	2007	EVEREST	20 countries^a^	2,085	AHF	7 days	Con + 30 mg/day of tolvaptan (*n* = 1,054)	Con + Placebo (*n* = 1,031)	[A]
Konstam et al. (19)	2007	NR (EVEREST Outcome)	20 countries^a^	4,133	AHF	8 days	Con + 30 mg/day of tolvaptan (*n* = 2,072)	Con + Placebo (*n* = 2,061)	[C], [E], [F], [G], [H], [I]
Gheorghiade et al. (20)	2004	ACTIV in CHF	Argentina, USA	158	AHF	7 days	Con + 30 mg/day of tolvaptan (*n* = 78)	Con + Placebo (*n* = 80)	[E], [F], [I]

[A], dyspnea relief within 24 h; [B] dyspnea relief within 48 h; [C] change in body weight within 48 h; [D] change in weight within 7 days; [E] edema reduction; [F] change in serum sodium level; [G] change in serum creatinine level; [H] mortality; and [I] rehospitalization.

^a^ 359 centers in 20 North American, South American, and European countries.

### 2.5 Statistical analysis

Data analysis was conducted using RevMan 5.4.1, with relative risk (RR), mean difference (MD), and 95% confidence interval (CI) adopted as influence measures. Under an incidental effect model, the Mantel–Haenszel method was utilized to analyze the dichotomous data. The I2 test determined the presence of heterogeneity, and the significance was set at *p* < 0.05. A random-effect model was used when *P* < 0.1 and I2 ≥ 50%. Additional subgroup examinations were performed to investigate the potential causes of statistical heterogeneity.

The NMA adopted both direct and indirect comparisons to estimate the relative efficacy of the different tolvaptan dosages. It is a method that allows for the simultaneous comparison of multiple interventions in a single study by incorporating both direct and indirect data from a network of studies. It generates the estimates of the comparative effects of any two interventions in the network and typically provides more accurate estimates compared to a single direct or indirect estimate. Additionally, it enables the estimation of the relative position and order of interventions (22). The analysis was conducted by utilizing the GEMTC package (Version 1.1-0) in the R programming language (The R Foundation for Statistical Computing, Vienna, Austria). For indirect therapy comparisons, the NMA was used based on hierarchical Bayesian models to compare the effects of distinct interventions. The Bayesian framework was based on the probability distribution of the model parameters, taking into account both the observed data and prior beliefs derived from external information regarding the parameter values. The importance of the differences between direct and indirect comparisons was imagined using contrast plots. The probability of treatment ranking for each dosage of tolvaptan was determined using the MetaInsight V4.0.2 Beta software. The results were then presented using the Litmus Rank-O-Gram surface under the cumulative ranking curve (SUCRA) (23).

A sensitivity examination was adopted to assess the impact of specific studies on the estimations. Each analysis was eliminated separately, and the results were recalculated to evaluate their strength. In addition, a leave-one-out analysis was adopted to perform multiple meta-analyses by excluding the effect size that might distort the total results (Supplementary S5). *P* < 0.05 was considered statistically significant.

## 3 Result

### 3.1 Literature search and included studies

**Figure 1:**
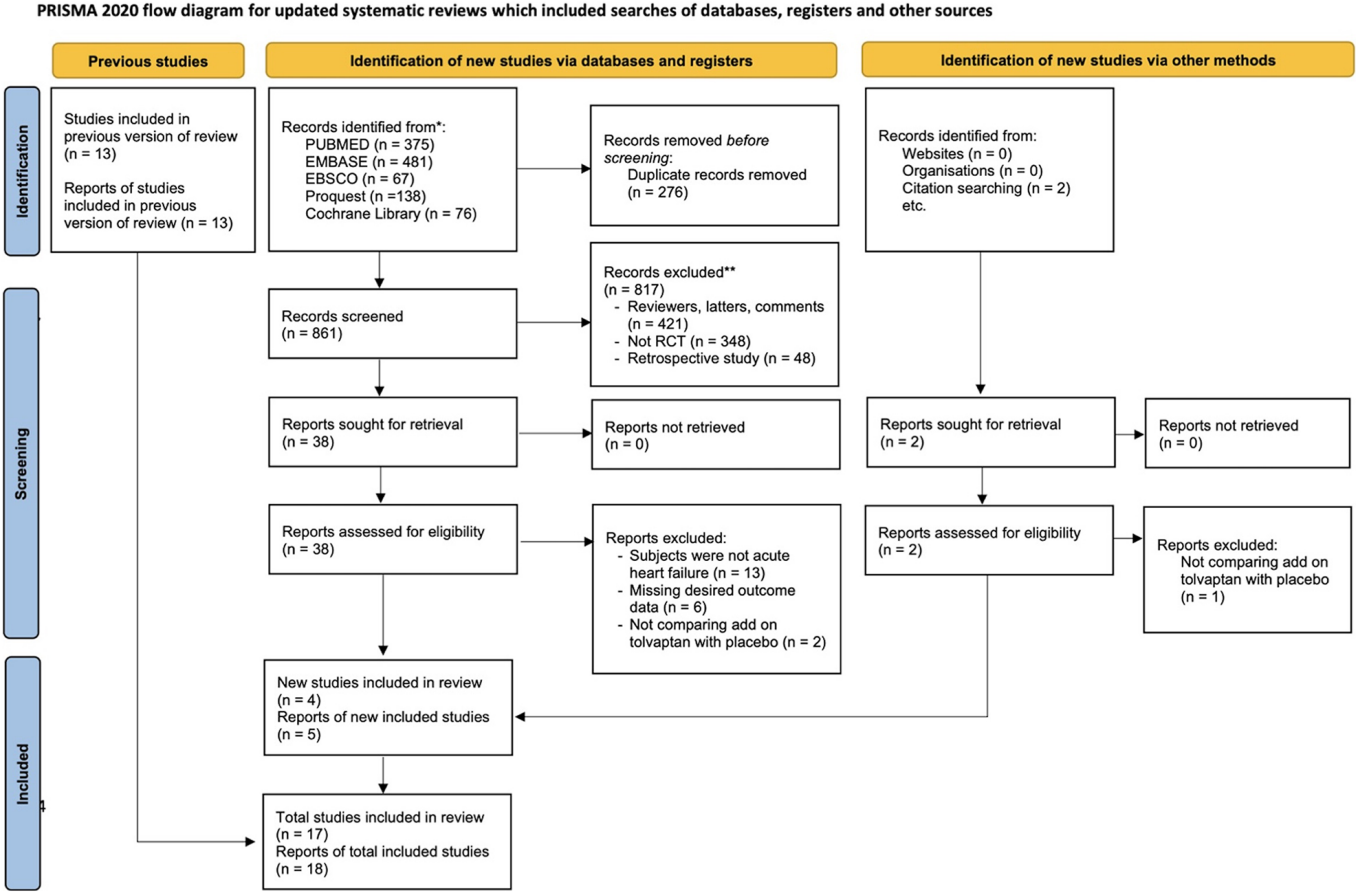
PRISMA flowchart showing the eligibility of studies for inclusion in the updated meta-analysis.

Figure 1 presents a diagram showing the study selection. A total of 1,137 studies were identified from PubMed (375), Embase (481), EBSCO (67), ProQuest (138), and Cochrane Library (76). After 276 duplicates were removed, 861 studies were subjected to screening. Among these, 817 papers were excluded during the title and abstract screening phase due to not meeting the inclusion standards. Following a thorough assessment of the full-text papers, 22 studies were excluded due to lack of AHF subjects, missing desired outcome data, and not comparing tolvaptan add-on therapy with the conventional therapy with placebo. In addition to the database search, a citation search was conducted for the included papers, resulting in the retrieval and assessment of two studies. Finally, 17 studies and 18 reports, which were published until 24 May 2023, were chosen for the meta-analysis based on the inclusion standards (3, 5–20).

### 3.2 Characteristics of the included studies and quality assessment

Table 1 summarizes the characteristics of the included studies, such as the first reviewer, publication year, study acronym, study country, sample size, study period, intervention type(s), and clinical outcome(s). The 17 RCTs and 18 records comprised studies shown in the USA, South America, Europe, Japan, Taiwan, India, and Thailand. The studies included a total of 9,988 patients with AHF, of whom 5,000 (50.06%) patients belong to the tolvaptan group and 4,988 (49.94%) patients belong to the control group.

### 3.3 Efficacy and safety indicators: clinical outcomes

This analysis compared dyspnea relief, changes in body weight, edema reduction, changes in sodium and creatinine levels, mortality, and rehospitalization associated with tolvaptan add-on and conventional therapies. Mortality and rehospitalization outcomes were associated with cardiovascular causes.

#### 3.3.1 Effect of tolvaptan add-on therapy on dyspnea relief

**Figure 2:**
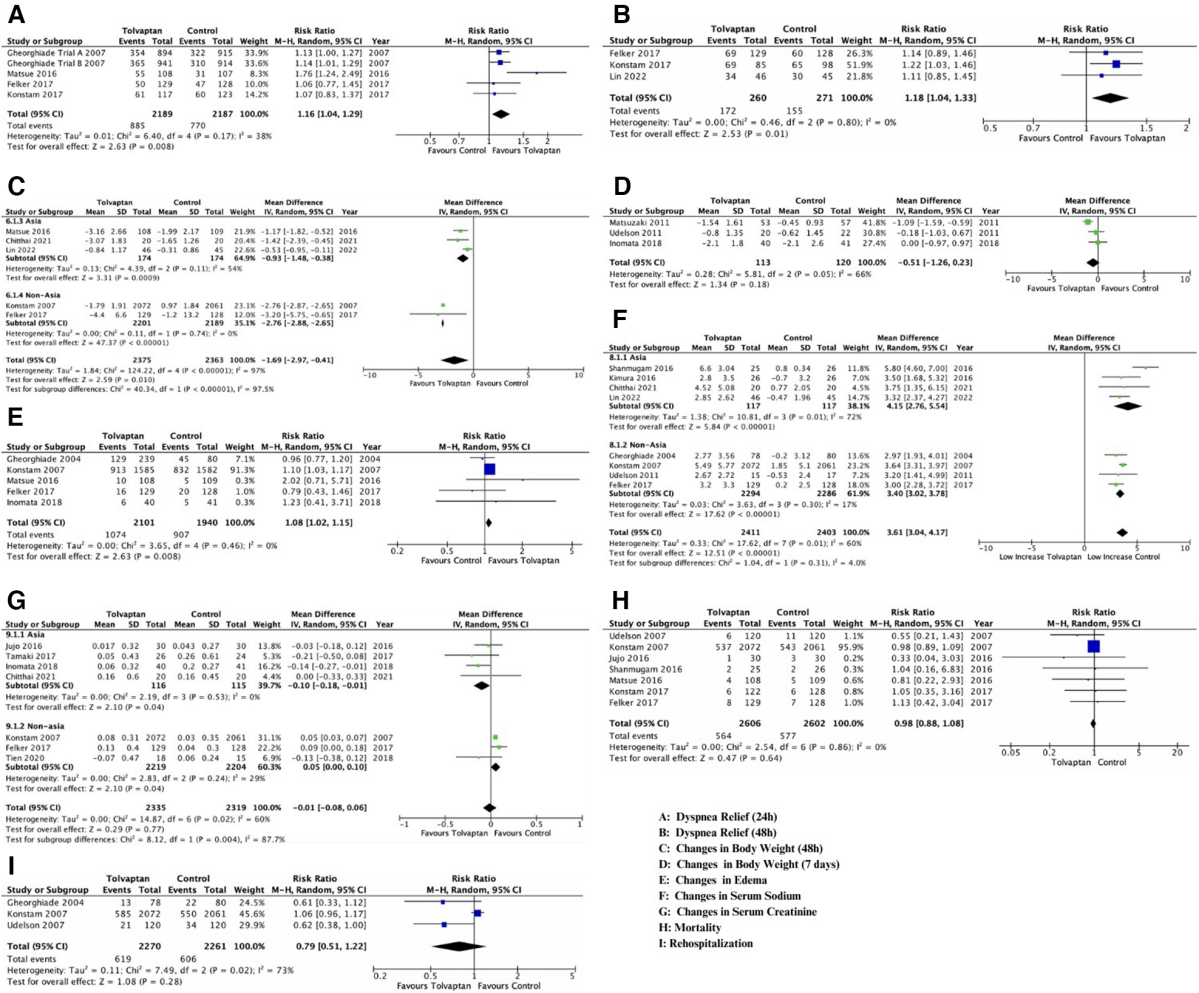
Forest plot depicting the effects of add-on tolvaptan on clinical outcomes. The effects of add-on tolvaptan on dyspnea relief within 24 h (**A**), dyspnea relief within 48 h (**B**), changes in body weight within 48 h (**C**), changes in body weight within 7 days (**D**), edema reduction (**E**), changes in serum sodium (**F**) and creatinine (**G**) levels, mortality (**H**), and rehospitalization (**I**).

The primary reason for hospitalization in HF patients was dyspnea. Five reports documented the changes in dyspnea within 24 h (Figure 2A), and three reports addressed the changes in dyspnea within 48 h (Figure 2B) using a Likert psychometric scale. No substantial heterogeneity was observed among all studies (dyspnea 24 h, *P* = 0.17, I2 = 38%; dyspnea 48 h, *P* = 0.80, I2 = 0%), and the analysis was performed using the random-effect model. The meta-analysis displayed a significant advancement in dyspnea in the tolvaptan group within 24 h [RR 1.16 (1.04, 1.29), *p* = 0.008] and 48 h [RR 1.18 (1.04, 1.33), *p* = 0.01].

#### 3.3.2 Effect of tolvaptan add-on therapy on reducing body weight within 48 h (BW 48 h) and 7 days (BW 7 days)

The aquaretic impact of the tolvaptan add-on therapy manifested in the mean body weight of HF patients. There was significant heterogeneity among the five analyses that examined both reduced body weight outcomes (BW 48 h, *P* < 0.001, I2 = 97%; BW 7 days, *P* = 0.05, I2 = 66%) (Figures 2C,D), and a subgroup analysis was conducted (year, location). Based on the subgroup analysis, the location appeared to influence the heterogenicity of BW 48 h outcomes. Therefore, the conclusion was drawn from these subgroups, and a random-effect model was adopted (Figure 2C). A substantial weight reduction within 48 h with tolvaptan compared to placebo was observed in both Asian and non-Asian subgroups [Asian group, MD −0.93 (−1.48, −0.38), *p* = 0.00; non-Asian group, MD −2.76 (−2.88, −2.65), *p* = 0.00]. However, the subgroup analysis for BW 7 days showed a significant heterogenicity, which might be influenced by the low number of included studies. The analysis showed no substantial weight reduction within 7 days with tolvaptan [MD−0.51 (−1.26, 0.23), *p* = 0.18] (Figure 2D).

#### 3.3.3 Influence of tolvaptan add-on therapy on reducing edema

Diuretics might benefit HF patients with pitting edema and no nutritional weight gain due to reduced urine production and excessive fluid retention. This analysis showed a significant reduction in edema [RR 1.08 (1.02, 1.15), *p* = 0.008)] with tolvaptan add-on therapy compared to the conventional therapy (Figure 2E). No important heterogeneity was observed among the analyses, and the random-effect model was used for the examination (edema, *P* = 0.46, I2 = 0%).

#### 3.3.4 Effect of tolvaptan add-on therapy on serum sodium and creatinine

Significant heterogeneity existed among studies that discussed both changes in serum sodium and creatinine (sodium, *P* = 0.01, I2 = 60%; creatinine, *P* = 0.02, I2 = 60%), thereby prompting a subgroup analysis. Both outcomes showed that location (non-Asia for serum sodium and Asia for serum creatinine) influenced heterogenicity. Conclusions were drawn from location subgroups, and the random-effect model was adopted (Figures 2F,G).

Among the four studies in the non-Asian subgroup (Figure 2F), a significant rise was observed in patients with tolvaptan [MD 3.40 (3.02, 3.78), *p* < 0.00001]. Based on the conveyed change among four studies in the Asian group (Figure 2G), a significant difference in the serum creatinine change was found between patients feted with tolvaptan vs. placebo [MD −0.10 (−0.18, −0.01), *p* = 0.04].

#### 3.3.5 Effect of tolvaptan add-on therapy on mortality and rehospitalization

No meaningful heterogeneity was observed in mortality studies (*P* = 0.86, I2 = 0%), while rehospitalization had a significant heterogeneity. Despite conducting a subgroup analysis, there was heterogeneity possibly influenced by the low number of included studies. The analysis adopted the random-effect model, and seven studies reported that there was no distinction in mortality [RR 0.98 (0.88, 1.08), *p* = 0.20] (Figure 2H). Additionally, there was no disparity in rehospitalizations [RR 0.79 (0.51, 1.22), *p* = 0.28, I2 73%] in patients treated with tolvaptan compared to placebo (Figure 2I).

### 3.4 NMA of the tolvaptan dosage effect subgroup

**Figure 3:**
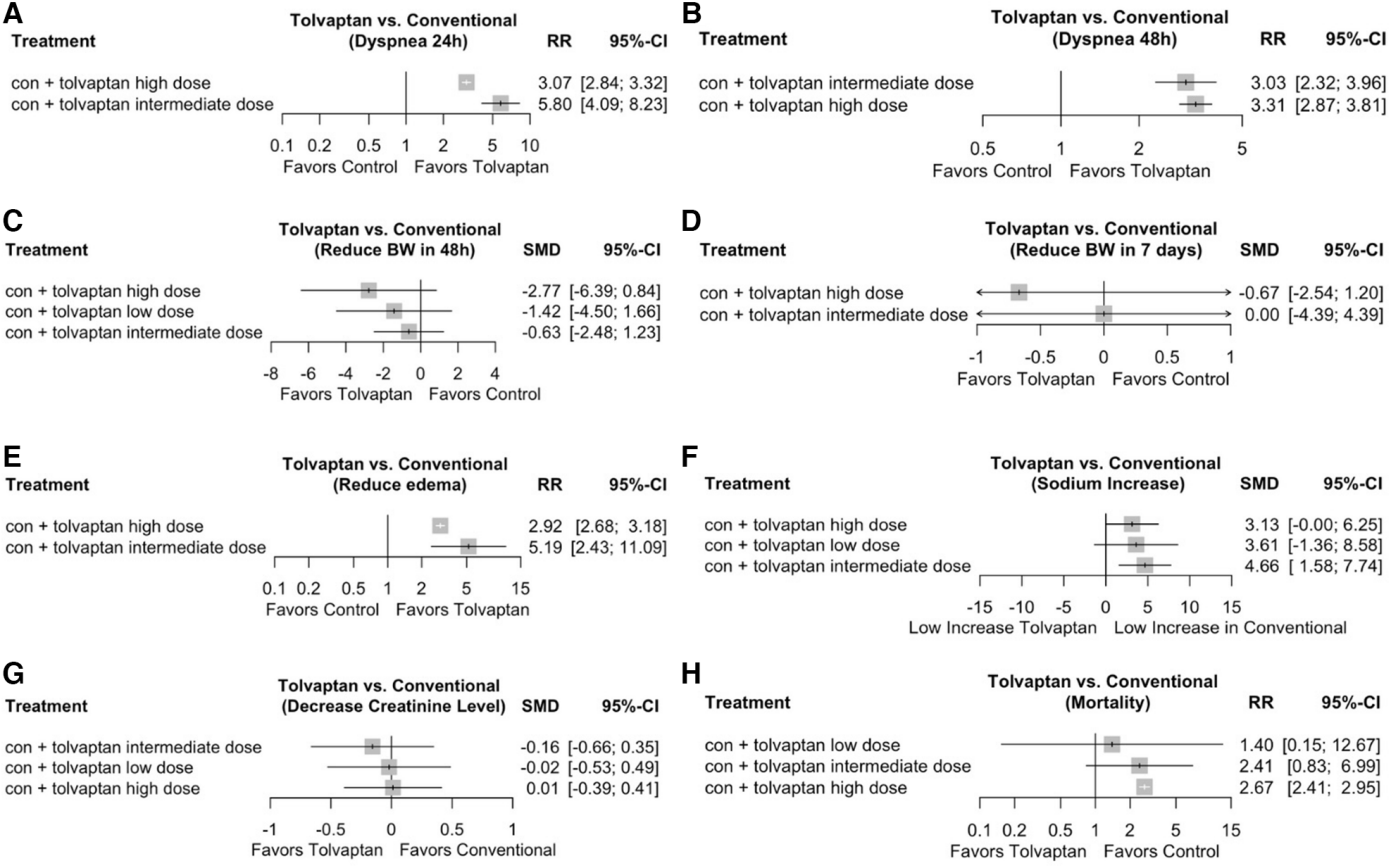
Network meta-analysis (NMA) on the tolvaptan dosage effect subgroup analysis. Contrast plot showing the dosage effect of each efficacy and safety indicator. (**A**) Network analysis contrast plot of dyspnea within 24 h; (**B**) network analysis contrast plot of dyspnea within ≥48 h; (**C**) network analysis contrast plot of reduced body weight within 48 h; (**D**) network analysis contrast plot of reduced body weight within 7 days; (**E**) network analysis contrast plot of edema; (**F**) network analysis contrast plot of serum sodium level; (**G**) network analysis contrast plot of serum creatinine level; and (**H**) network analysis contrast plot of mortality.

**Figure 4:**
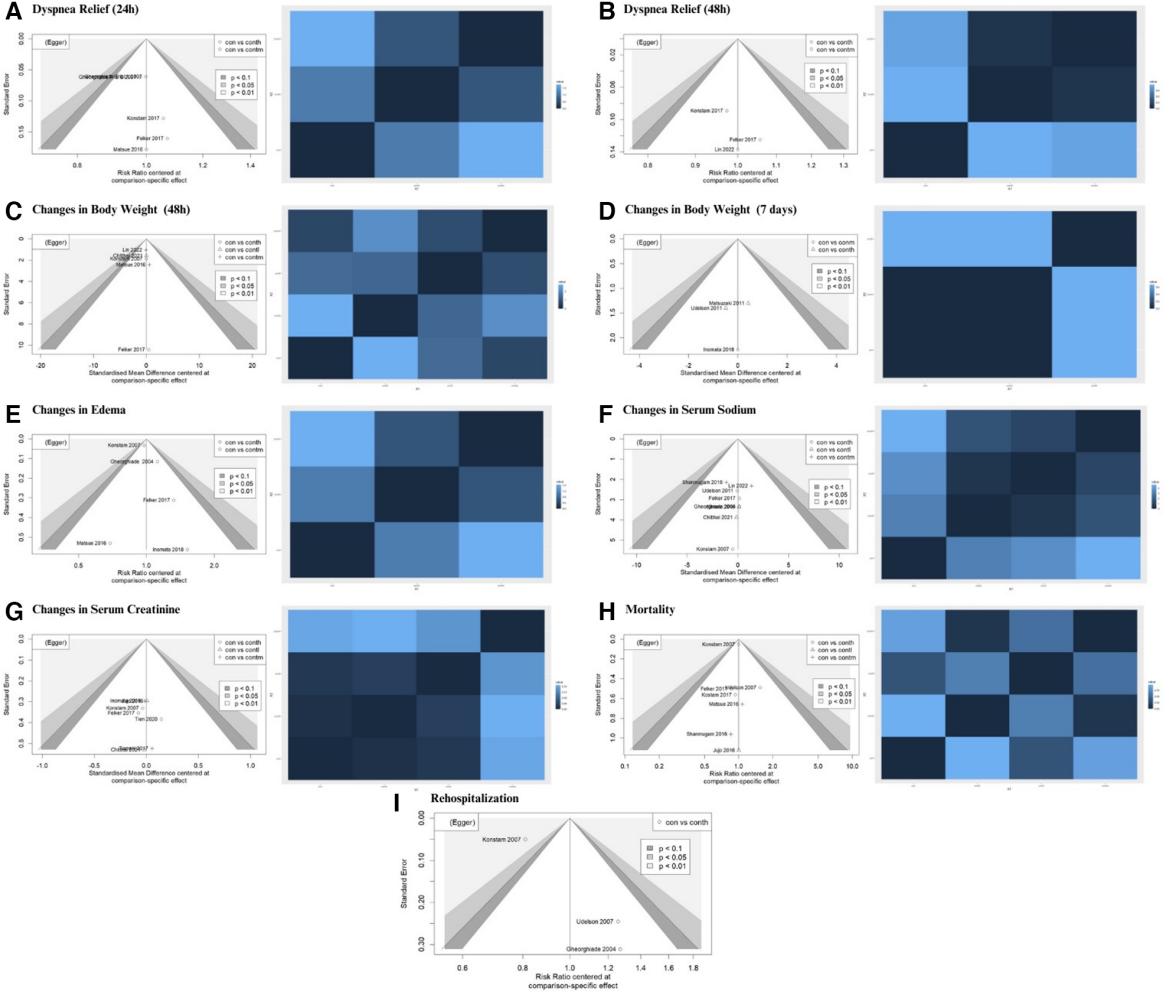
Contour-enhanced funnel plot and heat map for each efficacy and safety indicator.

The NMA was performed to compare the efficacy of the dosage effect by stratifying tolvaptan therapy classes into low dosage (7.5 mg/day), intermediate dosage (15 mg/day), and high dosage (30 mg/day). The analysis consisted of comparing three or more therapies, using both indirect comparisons across trials based on a standard comparator (conventional therapy) and natural comparisons of interventions (high dosage vs. intermediate dosage vs. low dosage) in RCTs. The choice of tolvaptan dosage was based on previous reviews and various protocols. Figure 3 presents the contrast plots reporting the risk ratio for each tolvaptan dosage and outcomes. A contour-enhanced funnel plot and a heat map were also included in the study (Figure 4). We provided a detailed overview of the rankings for different tolvaptan dosage methods based on the SUCRA probability. We assessed the rankings based on dyspnea relief within 24 h and 48 h, changes in body weight within 48 h and 7 days, edema reduction, changes in sodium level, changes in creatinine level, and mortality (Supplementary S6). Supplementary data (Supplementary S7, S8) also display the pooled estimates of the NMA and the network plots for each analysis.

### 3.5 Sensitivity analysis

Sensitivity analysis was performed for each efficacy and safety indicator. The leave-one-out analysis was adopted to perform multiple meta-analyses by excluding the effect size that might distort the total results. The leave-one-out forest plot also provided a vertical line at the effect size based on the complete set of studies (with no omission) to detect influential studies, as shown in Supplementary S5. Several reviews showed non-robust results, specifically in outcomes that had heterogeneity in the clinical and safety meta-analysis.

### 3.6 Publication bias

Publication bias was visualized using a contour-enhanced funnel plot (Figure 4). Integrating a contour-enhanced funnel plot and the Eager test (Supplementary S4) revealed that there was no significant publication bias in summarizing the impact of add-on tolvaptan.

## 4 Discussion

The meta-analysis showed that tolvaptan add-on therapy significantly alleviated dyspnea by reducing the volume overload. The tolvaptan group showed a substantial reduction in edema and short-term weight within 48 h, but this weight shift was temporary, without significant changes observed within 7 days. Additionally, the tolvaptan group showed an increase in serum sodium during hospitalization in non-Asian studies. Meanwhile, Asian studies suggested that tolvaptan add-on therapy could significantly reduce the incidence of WRF. The NMA of the tolvaptan dosage effect subgroup showed that the intermediate dosage of 15 mg might be considered the optimal dosage for the majority of the outcomes.

In AHF patients presenting with predominant symptoms of fluid overload and congestion, intravenous loop diuretics served as the primary therapy to enhance renal excretion of sodium and fluids, while also acting as a vasodilator to provide rapid relief of congestive symptoms (24). Nevertheless, the acknowledged drawbacks in both effectiveness and safety, such as electrolyte disturbances and renal impairment, compel us to escalate the dosage of diuretics to prevent further neurohormonal activation and overcome resistance to their diuretic effects. It is widely recognized that diuretic effects steadily diminish over successive days of loop diuretic treatment. Hence, it is advisable to promptly adjust diuretic therapies, such as combination therapy, to promote decongestion (25). Tolvaptan, an oral diuretic, competes with vasopressin at the distal nephron V2 receptor. It prevents aquaporin channel activation by blocking the V2 receptor–arginine vasopressin connection. This prevents water from being reabsorbed, resulting in the excretion of free water (26). In line with the observation of Kansara et al., the meta-analysis concluded that adding tolvaptan could greatly reduce volume excess, as evidenced by relieved dyspnea, weight reduction within 48 h, and reduced edema. This suggested that tolvaptan aided in achieving the therapy objectives of HF, such as decreasing congestion, after-load, and neurohormonal activation to enhance hemodynamics and symptoms, potentially reducing in-hospital events, rehospitalizations, and mortality while bypassing therapy toxicities (27).

A drawback of conventional diuretics was the potential development of resistance and adverse effects, including electrolyte abnormalities. The results among non-Asian studies supported by the EVEREST and a previous meta-analysis showed that tolvaptan add-on therapy greatly increased sodium levels compared to conventional therapy. Tolvaptan, a selective non-peptide oral agent rival of the vasopressin receptor, acted on the distal part of the nephron by inhibiting the V2 receptor, suppressing the action of the antidiuretic hormone for reabsorption of free water, thereby resulting in the excretion of diluted urine, and facilitating hypotonic diuresis without adversely affecting the elimination of electrolytes. Furthermore, the mechanism prevented the activation of the aquaporin system, reducing the ability of the kidneys to reabsorb water and leading to an increase in sodium levels in the blood and excretion of water in urine (3, 28).

WRF, a prevalent comorbidity found in AHF patients (29), was described as an increase in creatinine level of ≥0.3 mg/dl in the first 5 days compared to the baseline at hospital admission (30). Traditionally, WRF represented the gold standard for assessing acute kidney failure in patients with AHF (31). This analysis showed that tolvaptan add-on therapy correlated with a more inferior incidence of WRF compared to conventional therapy, as evidenced by a significant disparity in creatinine change between the two levels in Asian studies. Loop diuretics for decongestion may cause WRF by rapidly decreasing blood volume. Stimulation of the RAA and sympathetic nerve systems reduces renal perfusion and glomerular filtration pressure, causing WRF (32). Therefore, the decreased occurrence of WRF can be related to the reduced dosage of loop diuretics, which is assisted by the aquaresis caused by tolvaptan.

Nowadays, a range of therapeutic drugs is employed as standard therapy for HF. The effects of tolvaptan on mortality and morbidity in patients with HF have not been thoroughly explained, unlike ACE-I and beta-blockers, which have been established as medications with a Class I recommendation and A level of evidence (33). This study showed that add-on tolvaptan did not reduce rehospitalization and mortality of patients with HF during therapy, consistent with a previous meta-analysis. While tolvaptan might not reduce long-term mortality, it offers an opportunity for the administration of diuretics at lower dosages, thereby mitigating electrolyte disturbances and enhancing patient safety (34).

A different therapy for AHF utilizes a simultaneous nephron blockade with thiazide diuretics and mineralocorticoid receptor antagonists to allow decongestant use. The addition of these two categories of medications is intended to augment the impact of loop diuretics. Nevertheless, a large-scale clinical trial has systematically assessed the efficacy of combining diuretics. However, alternative combination therapy with diuretics, such as nesiritide or renal dosage dopamine, fails to enhance decongestants or renal function in AHF patients (35). On the other hand, there is a scarcity of data on the effectiveness of tolvaptan when used independently. The first study that examined the possibility of using tolvaptan as a substitute for furosemide was RCT by Udelson et al. (16), which revealed that tolvaptan, as monotherapy or in combination with loop diuretics, increased the urine volume and reduced the body weight of HF patients without changes in the blood electrolyte values. Several guidelines have endorsed the utilization of tolvaptan in patients with AHF (35–38). The 2021 European Society of Cardiology (ESC) Guidelines explicitly state that the therapy of dilutional hyponatremia caused by HF should revolve around controlling the water intake using tolvaptan, a vasopressin antagonist (35). Other Asian country guidelines recommend tolvaptan as a supplementary therapy for AHF patients who continue to experience congestion without distinguishing sodium levels (36–38).

Several clinical outcomes (changes in BW within 48 h and sodium and creatinine levels) suggested that location (non-Asia and Asia) influenced heterogenicity. This might be associated with current (epi)genome-wide connection analyses and HF. Furthermore, it has been identified that genes, metabolites, and pathways are associated with cardiovascular disease traits (39). Scheen et al. (40) reported that pharmacogenomics showed potential in individualized medicine treatment based on genetic and genomic data, thereby contributing to improvements in precision drugs and personalized drug therapy.

To further explore the relationship between tolvaptan dosage and various indicators, the NMA was conducted to compare low, intermediate, and high dosages. The choice of dosage was based on previous studies and consensus. The NMA resulted in diverse results, suggesting that high-dosage tolvaptan tended to have the highest likelihood of effectiveness in relieving dyspnea within 48 h and reducing BW within 48 h and 7 days. Meanwhile, the intermediate dosage indicated the highest probability of effectiveness in relieving dyspnea within 24 h, reducing edema, increasing sodium levels, and lowering the incidence of WRF. Low dosage only showed the best efficacy in mortality outcomes. The NMA findings were consistent with prior meta-analyses and guidelines, indicating that the addition of tolvaptan at intermediate dosages had a protective effect against WRF, but not at high dosages. The recommended initial dose of tolvaptan for general AHF patients is 7.5 mg–15 mg (36, 37, 41). Furthermore, multiple prior RCTs indicate that the occurrence rates of dry mouth, dehydration, and developing hypernatremia were higher in the 30 and 45 mg/day dose groups compared to the 15 mg/day dose group (13, 18). Lower starting dosages of tolvaptan are advised, especially for elderly individuals who are more prone to developing hypernatremia (42). Thus, for AHF add-on therapy, an intermediate dosage of tolvaptan, which is 15 mg/day, may be optimal.

## 5 Strengths and limitations

The NMA had several strengths distinct from other meta-analyses. First, a comprehensive search across multiple databases in all languages was conducted, minimizing the chance of overlooking significant studies. Second, subgroup analysis was adopted by grouping studies based on location and year, addressing heterogeneity in various analyses. Third, the NMA was conducted to determine the optimal dosage. The analysis independently assessed the efficacy of each dosage instead of combining multiple dosages into a single therapy group. Finally, sensitivity analysis was performed using the leave-one-out method, facilitating the identification of influential studies and assessing the robustness, particularly in outcomes showing heterogeneity.

This study has several limitations. First, the lack of details regarding conventional therapy, variations in diuretic dosages, and follow-up durations could potentially impact the results. Second, while analyzing the weight change within 7 days and rehospitalization, it was discovered that the investigations had a very high heterogenicity, and subgroup examination with further parameters was precluded due to insufficient data. Third, our study did not provide patient-centered outcomes, such as improvements in the quality of life or functional status. In addition, there are no long-term outcomes that compare tolvaptan's efficacy across different patient demographics in a specific area. These outcomes may provide a more thorough perspective on tolvaptan's benefits and a demographic overview of the research findings. Unfortunately, there is currently no original or primary study that incorporates these outcomes. Further research can be conducted to explore patient-centered outcomes and investigate specific areas, such as performing original research on each continent. Finally, the disparity in the number of studies that used intermediate- and low-dosage tolvaptan compared to high dosage could potentially influence the NMA results.

## 6 Conclusion

In conclusion, the tolvaptan add-on therapy for AHF patients proved beneficial for the short-term relief of dyspnea, initial weight reduction, edema reduction, increased sodium levels, and a reduced incidence of WRF; however, it did not lead to a significant benefit in lowering rehospitalization rates and mortality. The results suggested that an intermediate dosage of tolvaptan (15 mg/day) could be considered the optimal dosage for the majority of the outcomes.

## Data Availability

The original contributions presented in the study are included in the article/**Supplementary Material**, further inquiries can be directed to the corresponding author.
