# Supplementary material for: The role of tolvaptan add-on therapy in patients with acute heart failure: a systematic review and network meta-analysis
Source: Front Cardiovasc Med. 2024 May 30;11:1367442. doi: 10.3389/fcvm.2024.1367442 (PMC11169583; doi:10.3389/fcvm.2024.1367442)
Supplement: Supplementary file 2 [file Datasheet1.zip › Data Sheet 1_v1/Supplementary 1.DOCX]

**Supplementary 1.** Search Strategy.

| Database | Keywords | Results (Number of Studies) |
| --- | --- | --- |
| PubMed | ("acute heart failure"[All Fields] OR "heart failure"[MeSH Terms]) AND ("tolvaptan"[MeSH Terms] OR "samsca"[All Fields] OR "opc 41061"[All Fields]) | **375** |
| EMBASE | (TI acute heart failure OR AB acute heart failure OR TI heart failure OR AB heart failure) AND (TI tolvaptan OR AB tolvaptan OR TI samsca OR AB samsca OR TI opc 41061 OR AB opc 41061) | 481 |
| EBSCO | (TI acute heart failure OR AB acute heart failure OR TI heart failure OR AB heart failure) AND (TI tolvaptan OR AB tolvaptan OR TI samsca OR AB samsca OR TI opc 41061 OR AB opc 41061) | 67 |
| Proquest | (ti ("acute heart failure") OR ab ("acute heart failure") OR ti ("heart failure") OR ab ("heart failure")) AND (ti ("tolvaptan") OR ab ("tolvaptan") OR ti ("samsca") OR ab ("samsca") OR ti ("opc 41061") OR ab ("opc 41061")) | 138 |
| Cochrane Library | (TI acute heart failure OR AB acute heart failure OR TI heart failure OR AB heart failure) AND (TI tolvaptan OR AB tolvaptan OR TI samsca OR AB samsca OR TI opc 41061 OR AB opc 41061) | 76 |
| Total |  | 1137 |
