# Supplementary material for: The role of tolvaptan add-on therapy in patients with acute heart failure: a systematic review and network meta-analysis
Source: Front Cardiovasc Med. 2024 May 30;11:1367442. doi: 10.3389/fcvm.2024.1367442 (PMC11169583; doi:10.3389/fcvm.2024.1367442)
Supplement: Supplementary file 2 [file Datasheet1.zip › Data Sheet 1_v1/Supplementary 2.DOCX]

## Supplementary 2

| **Item** | **Definition** |
| --- | --- |
| **Population** | Patients with acute heart failure |
| **Intervention** | Conventional Therapy + Add on Tolvaptan Therapy |
| **Comparator** | Conventional Therapy + Placebo |
| **Outcome** | Dyspnea Relief within 24h; Dyspnea Relief within 48h; Edema Reduction; Change in Weight up tp 48h; Change in Weight up to 7 days, Change in Serum Sodium, Change in Serum Creatinine, Mortality, Rehospitalization |
| **Study** | Randomized Control Trial |
