# Supplementary material for: The role of tolvaptan add-on therapy in patients with acute heart failure: a systematic review and network meta-analysis
Source: Front Cardiovasc Med. 2024 May 30;11:1367442. doi: 10.3389/fcvm.2024.1367442 (PMC11169583; doi:10.3389/fcvm.2024.1367442)
Supplement: Supplementary file 2 [file Datasheet1.zip › Data Sheet 1_v1/Supplementary 3.DOCX]

## Supplementary 3. Risk bias graph (A) and summary (B): review authors’ judgments about each domain for each included study.


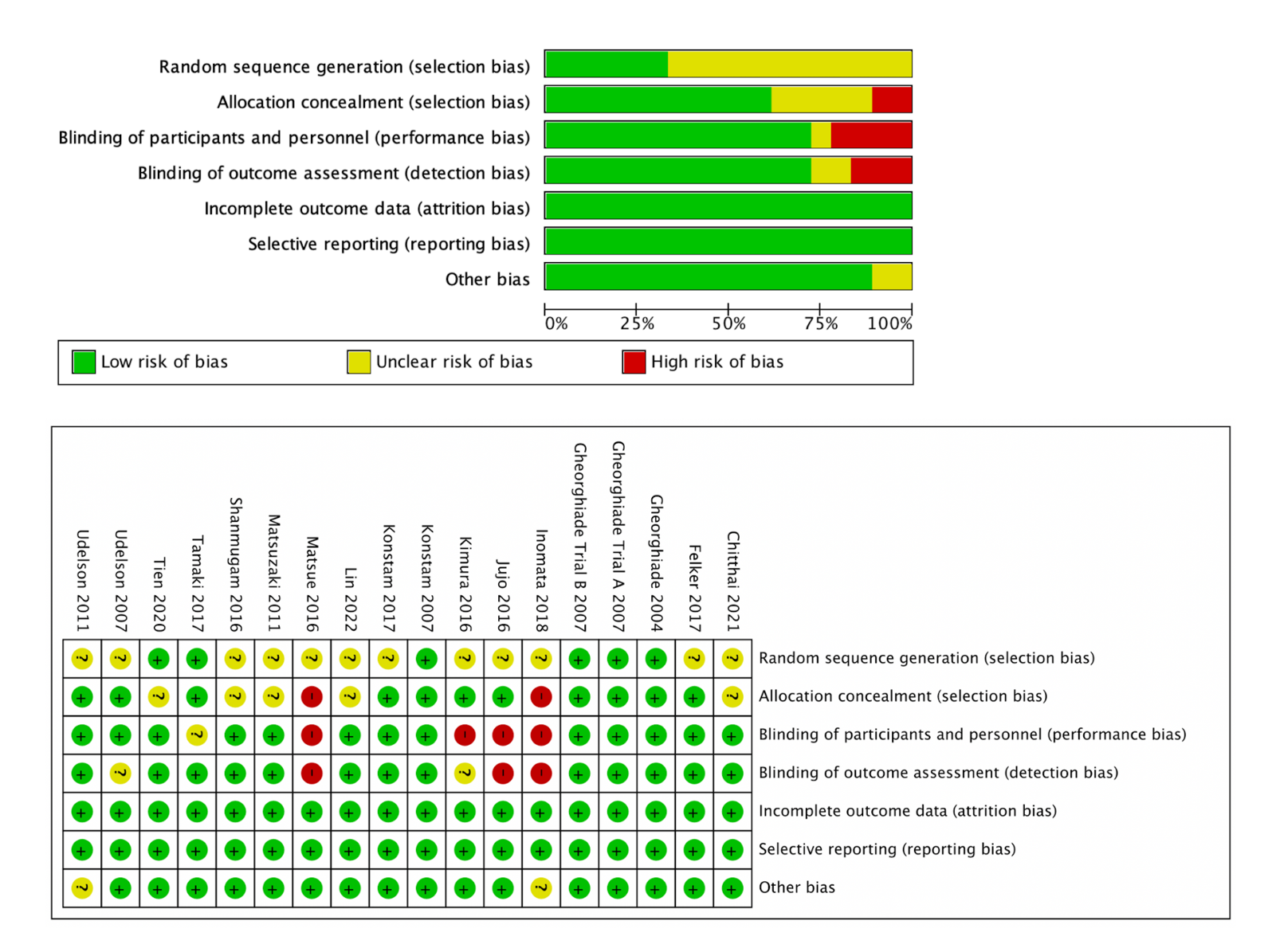


(A)

(B)
