# Supplementary material for: The role of tolvaptan add-on therapy in patients with acute heart failure: a systematic review and network meta-analysis
Source: Front Cardiovasc Med. 2024 May 30;11:1367442. doi: 10.3389/fcvm.2024.1367442 (PMC11169583; doi:10.3389/fcvm.2024.1367442)
Supplement: Supplementary file 2 [file Datasheet1.zip › Data Sheet 1_v1/Supplementary 4.DOCX]

## Supplementary 4. Egger test.

1. **
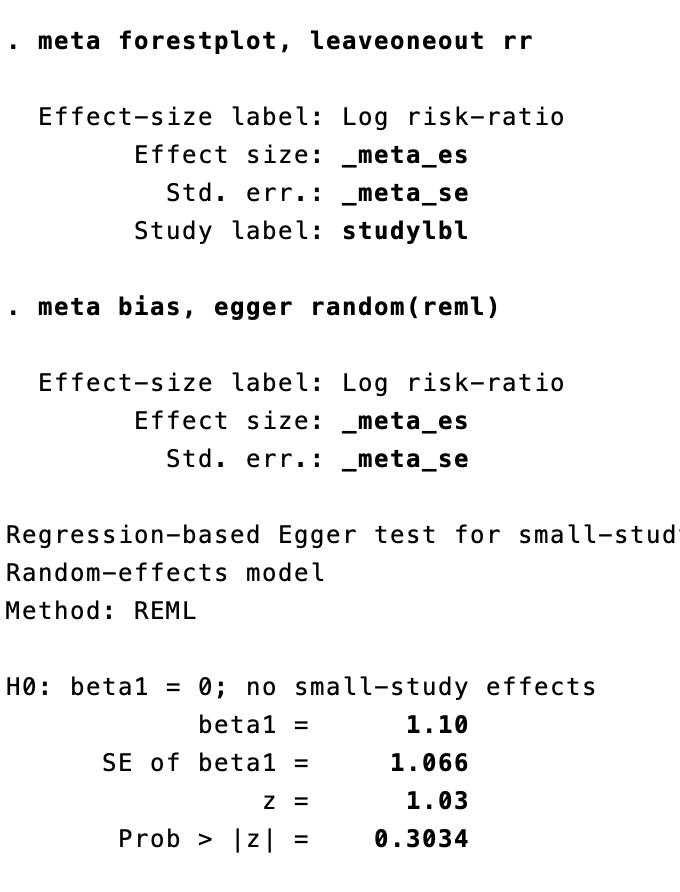
Dyspnea Relief within 24h.**
2. **
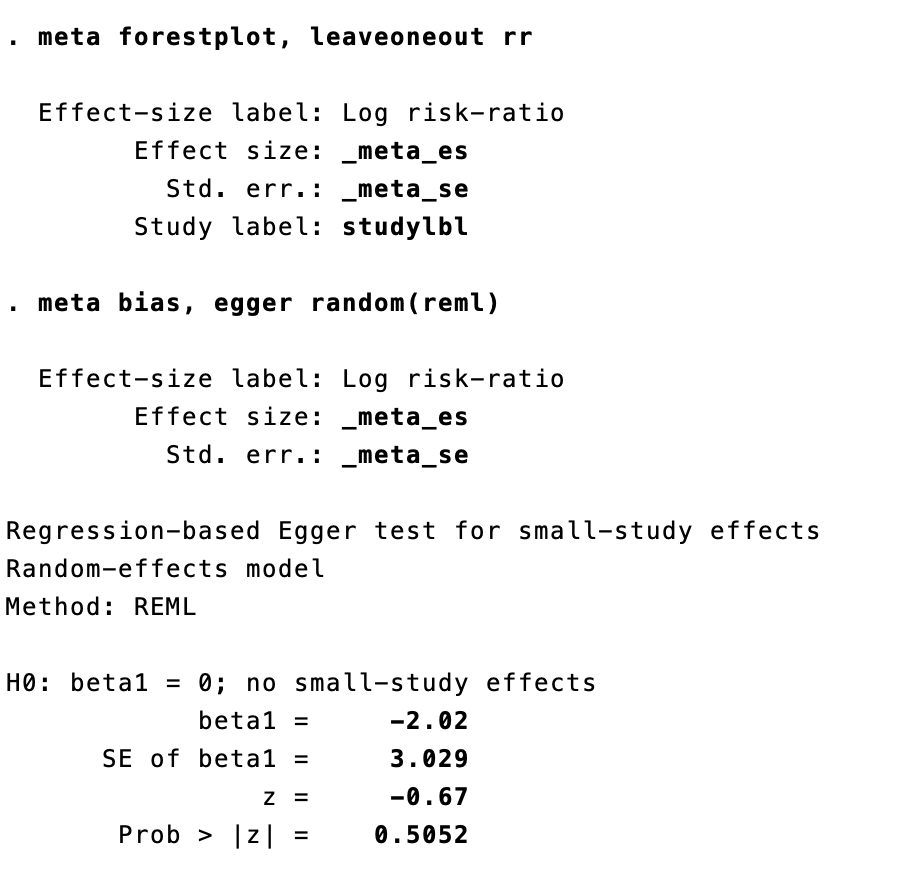
Dyspnea Relief within 48h.**
3. **
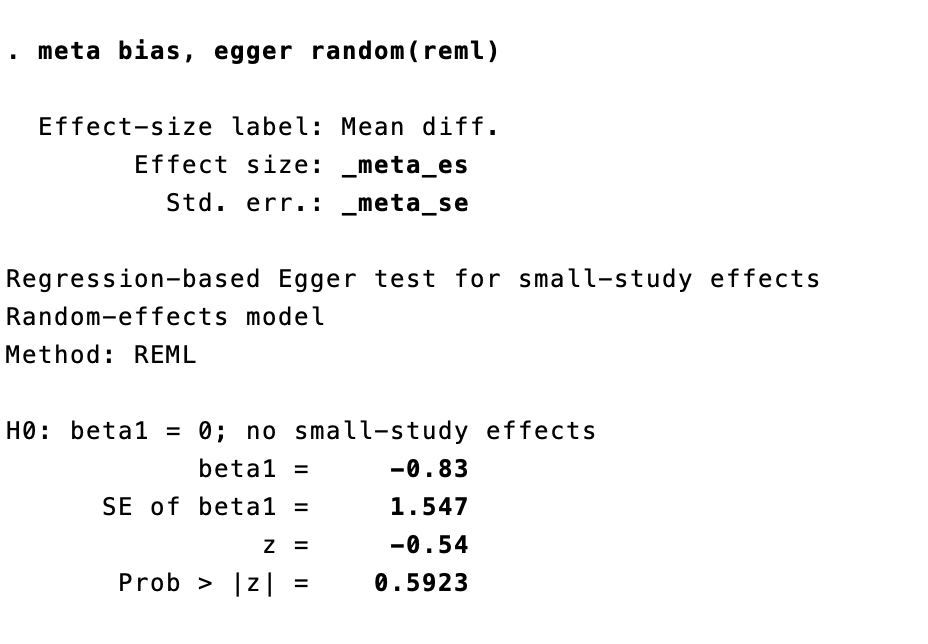
Change in Weight up to 48h.**
4. **
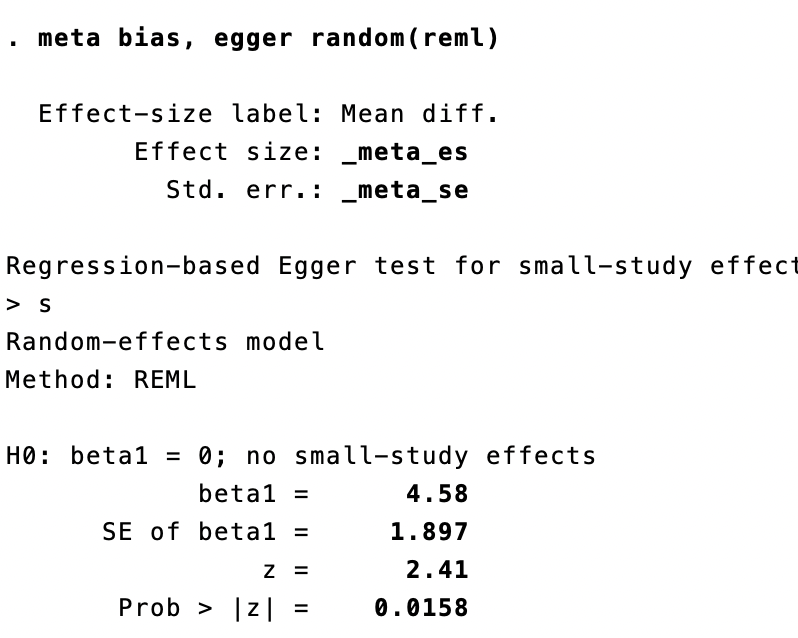
Change in Weight up to 7 days.**
5. **
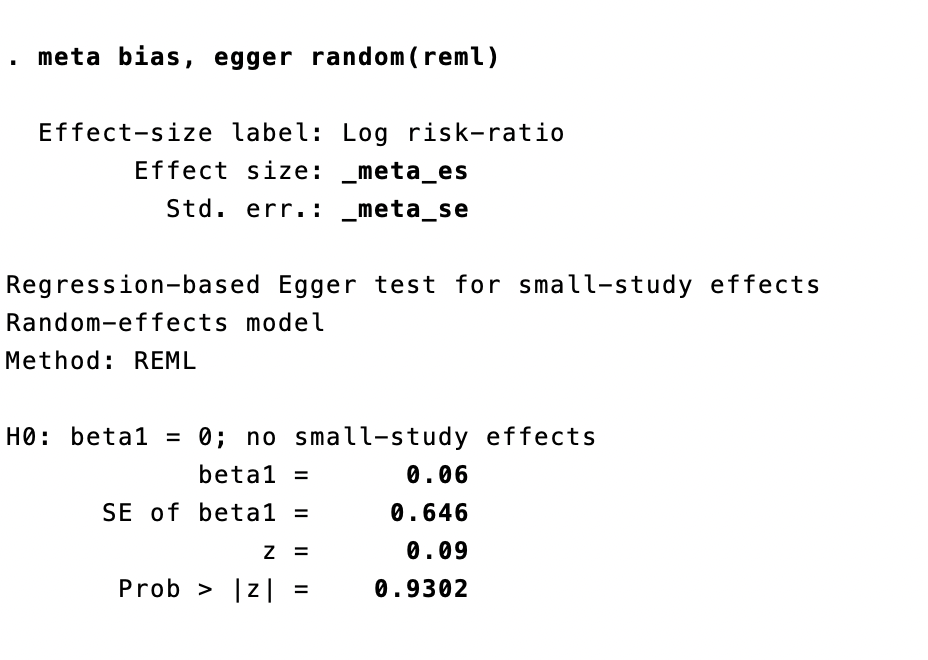
Edema Reduction.**
6. **
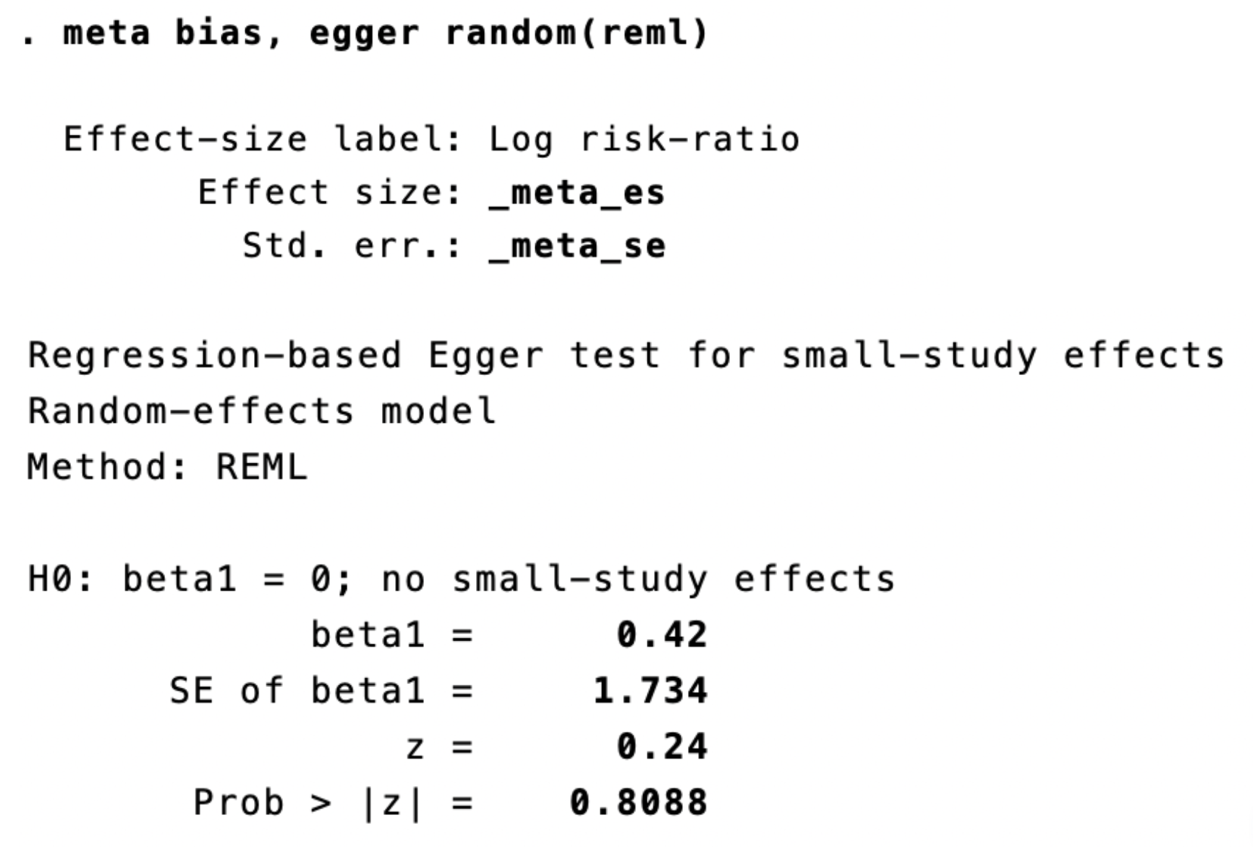
Change in Serum Sodium.**
7. **
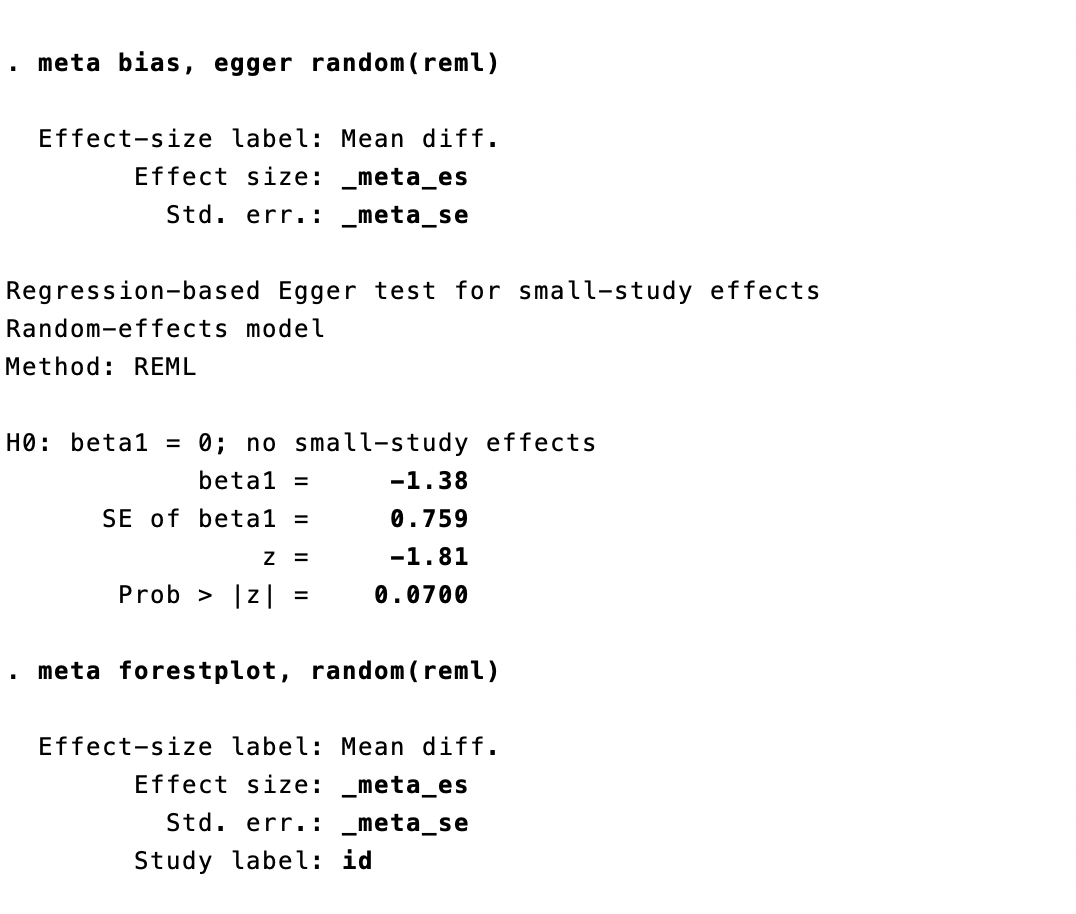
Change in Serum Creatinine.**
8. **
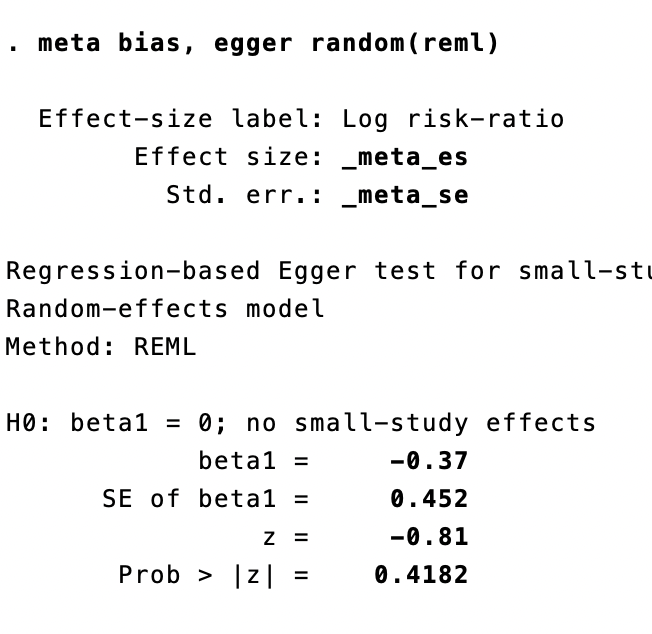
Mortality.**
9. **
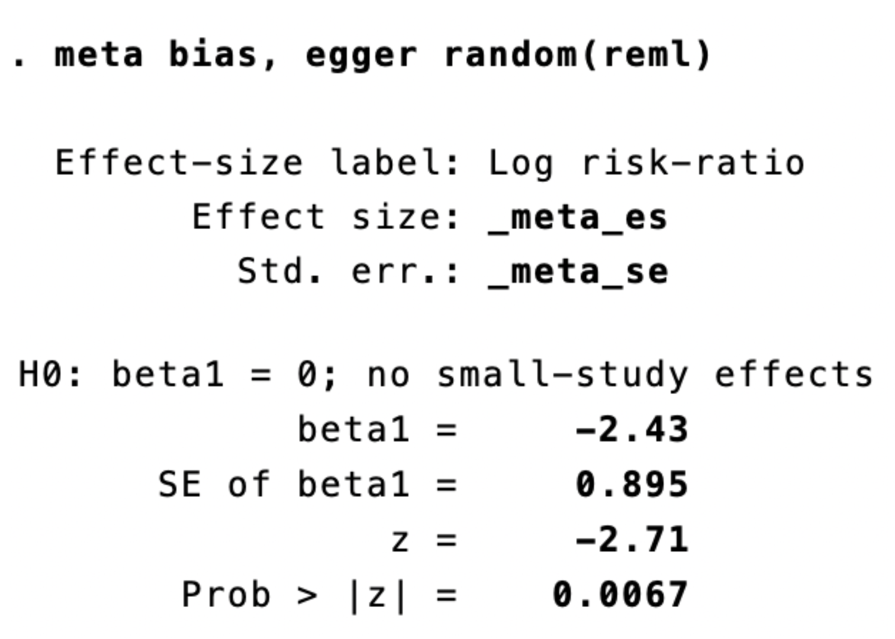
Rehospitalization.**
