# Supplementary material for: The role of tolvaptan add-on therapy in patients with acute heart failure: a systematic review and network meta-analysis
Source: Front Cardiovasc Med. 2024 May 30;11:1367442. doi: 10.3389/fcvm.2024.1367442 (PMC11169583; doi:10.3389/fcvm.2024.1367442)
Supplement: Supplementary file 2 [file Datasheet1.zip › Data Sheet 1_v1/Supplementary 5.DOCX]

## Supplementary 5. Leave-one-out Sensitivity Analysis. Forest plot Results of leave-one-out method in sensitivity analysis. The three vertical dotted lines denote the pooled random effect of RR or mean difference and 95% CI, while the solid vertical line shows the RR of 1. The horizontal lines and the circles indicate the RRs or mean differences and 95% CIs applying the leave-one-out method.


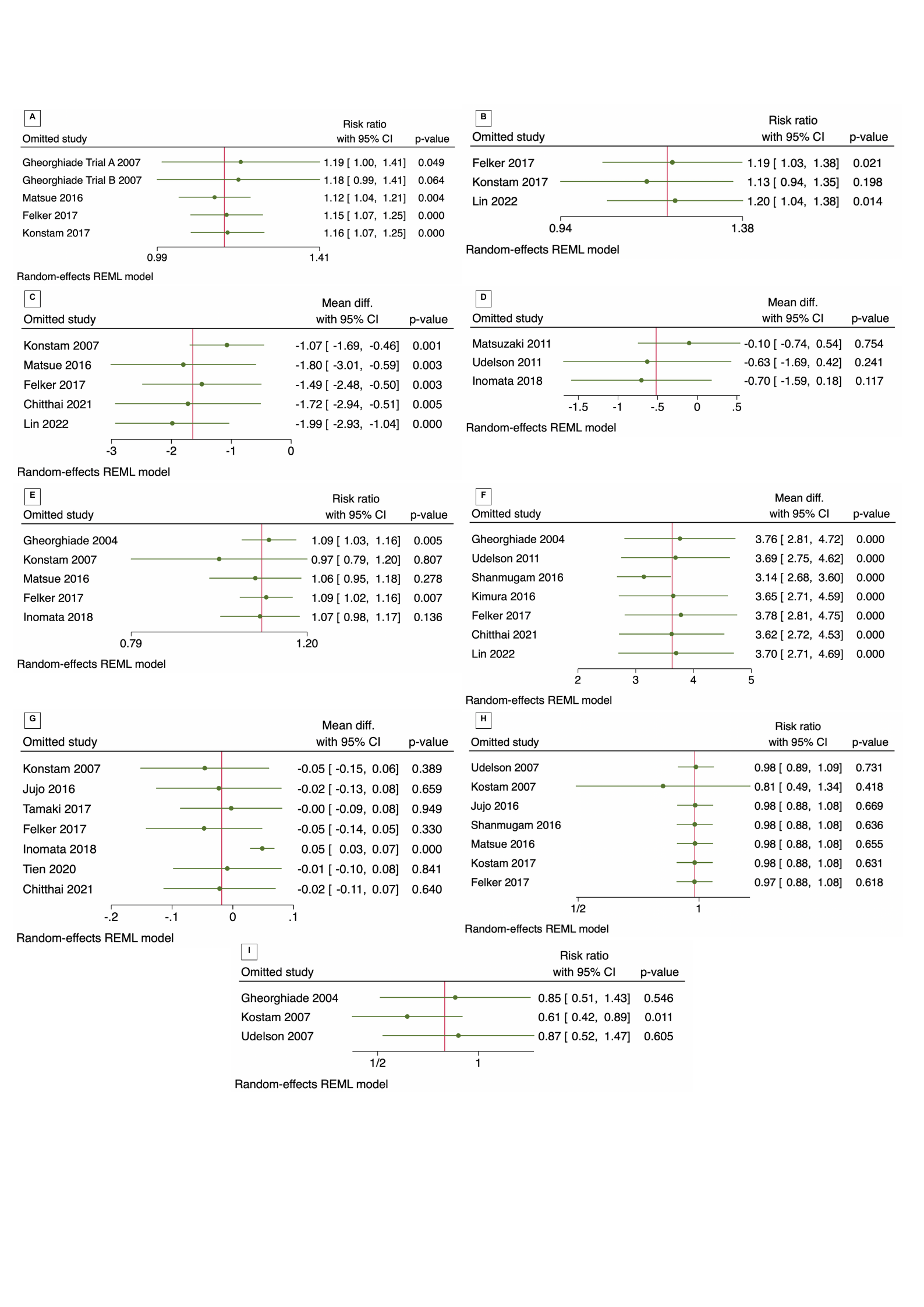


***** A: leave-one-out forest of dyspnea 24 hours; B: leave-one-out forest of dyspnea ≥ 48 hours; C: leave-one-out forest of reduce body weight up to 48h; D: leave-one-out forest of reduce body weight up to 7 days; E: leave-one-out forest of edema ; F: leave-one-out forest of serum sodium of ; G: leave-one-out forest of serum creatinine; H: leave-one-out forest of mortality; I: leave-one-out forest of rehospitalization;
