# Supplementary material for: The role of tolvaptan add-on therapy in patients with acute heart failure: a systematic review and network meta-analysis
Source: Front Cardiovasc Med. 2024 May 30;11:1367442. doi: 10.3389/fcvm.2024.1367442 (PMC11169583; doi:10.3389/fcvm.2024.1367442)

## Supplementary 6.

**Litmus Rank-O-Gram**. The effectiveness and safety results of Tolvaptan's doses are ranked using the surface under the cumulative ranking curve (SUCRA).

- Dyspnea Relief within 24h.


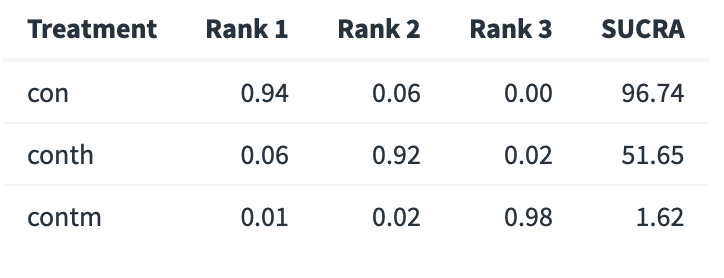

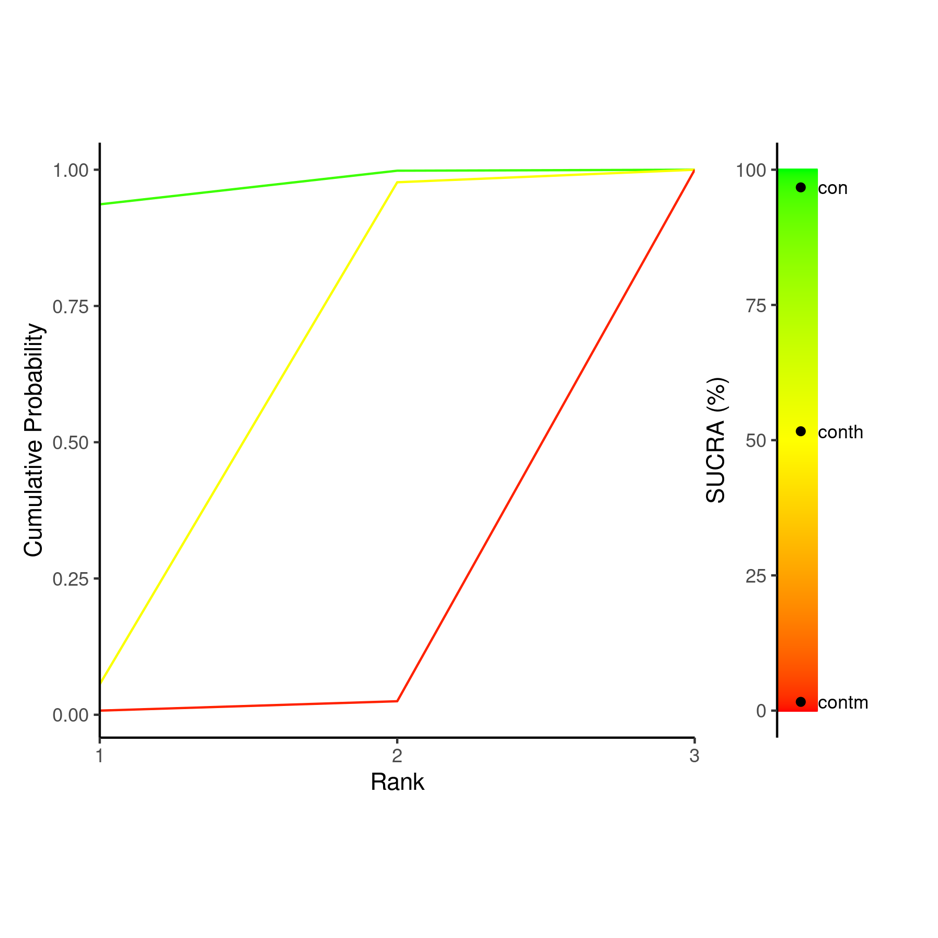


- Dyspnea Relief within 48h.


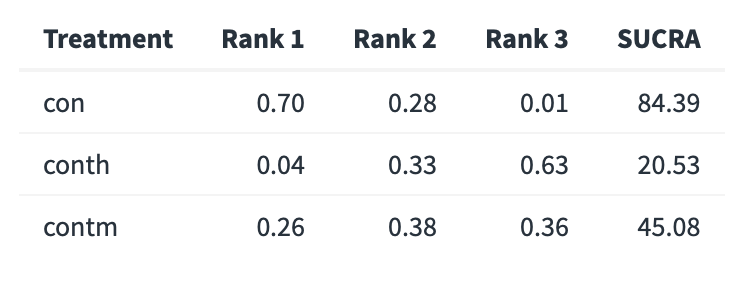

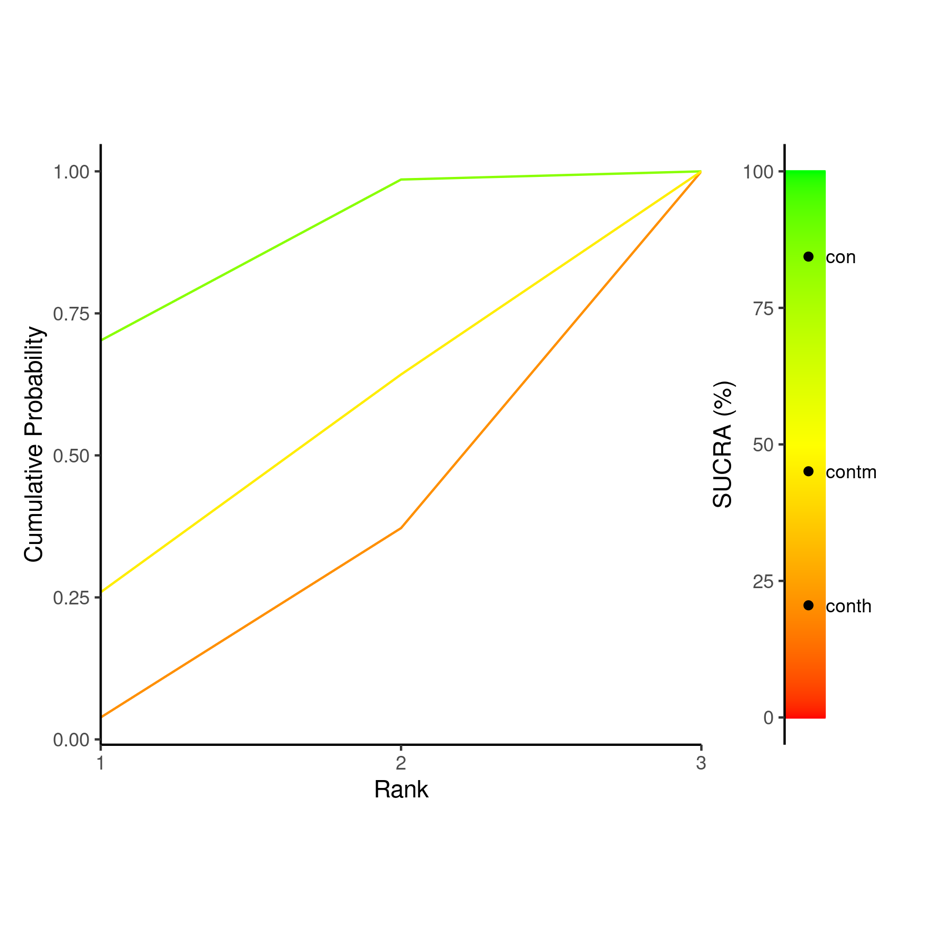


- Change in Weight up to 48h.


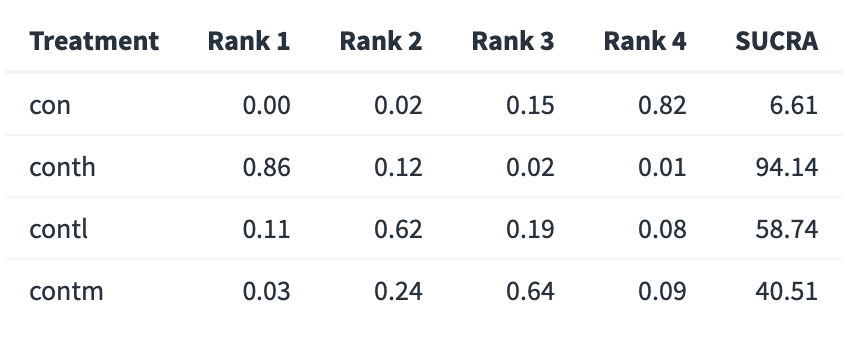

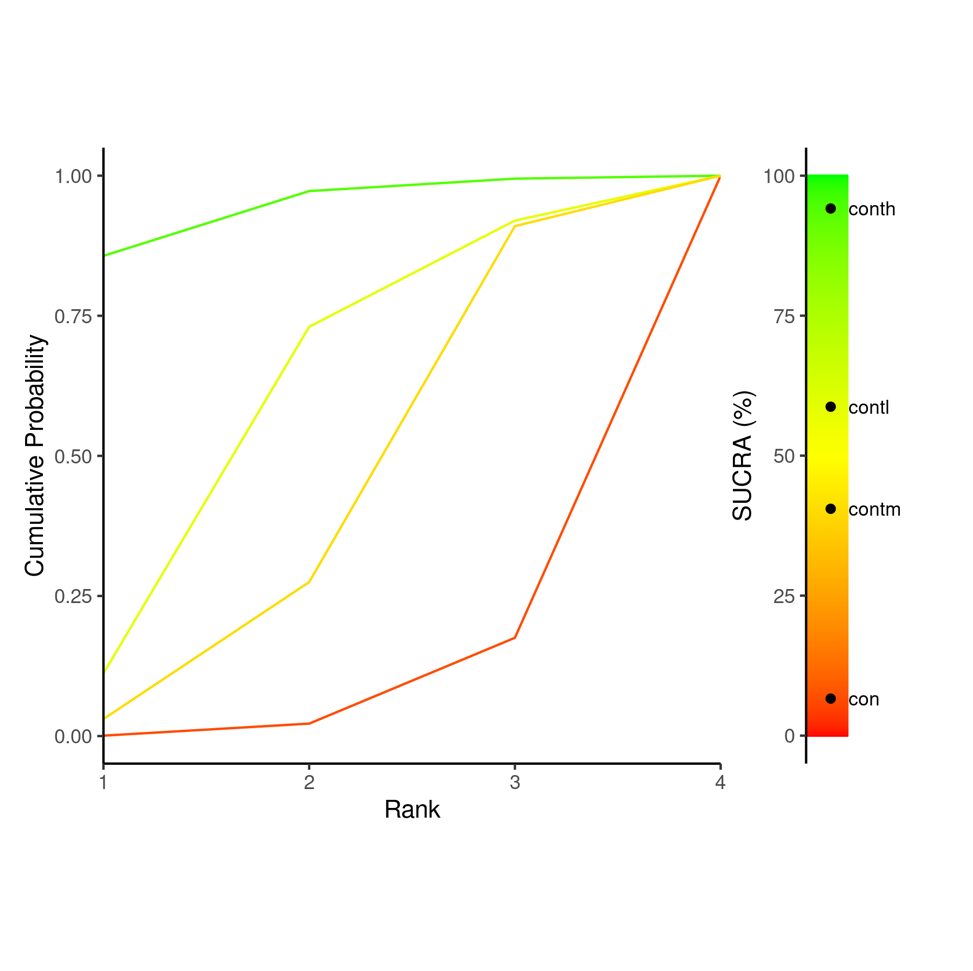


- Change in Weight up to 7 days.


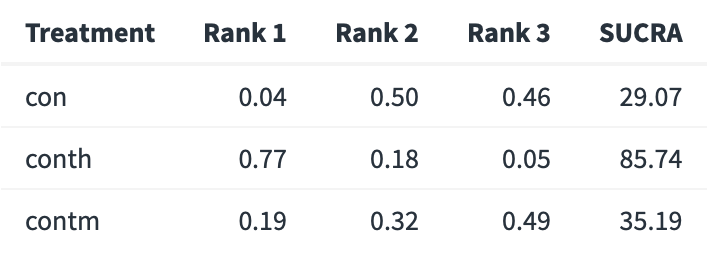

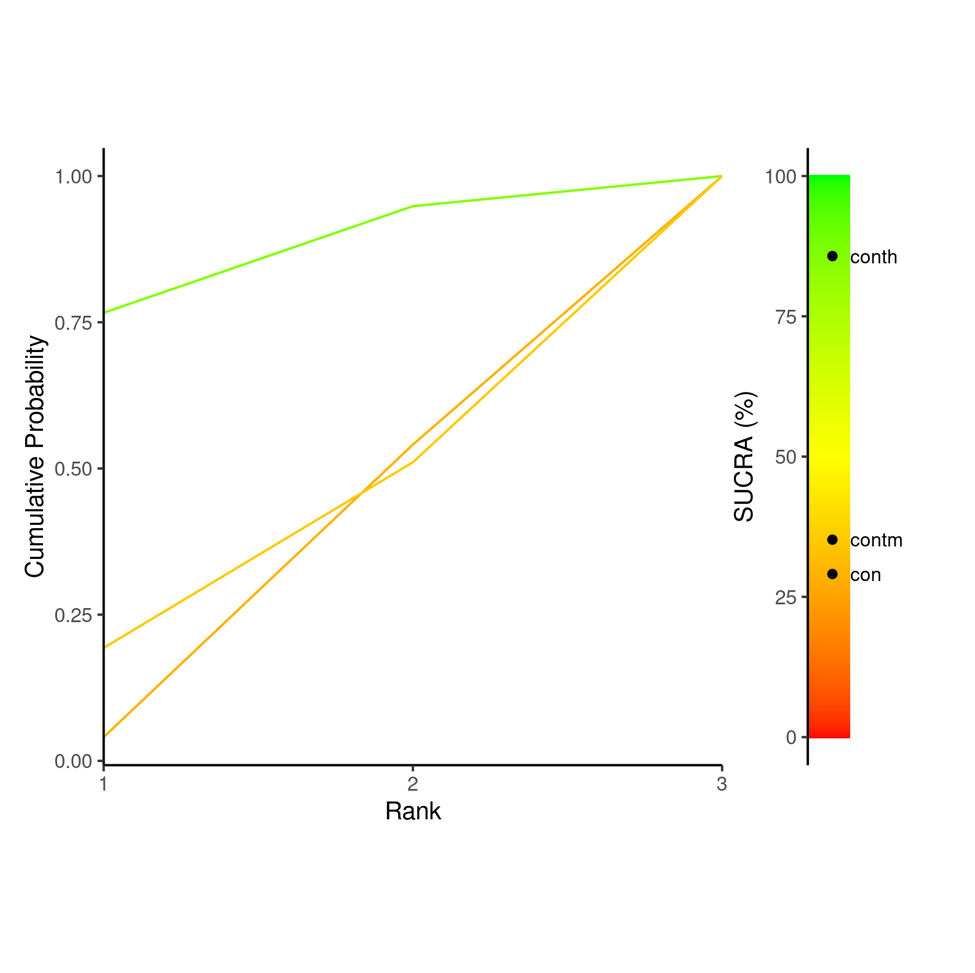


- Edema Reduction.


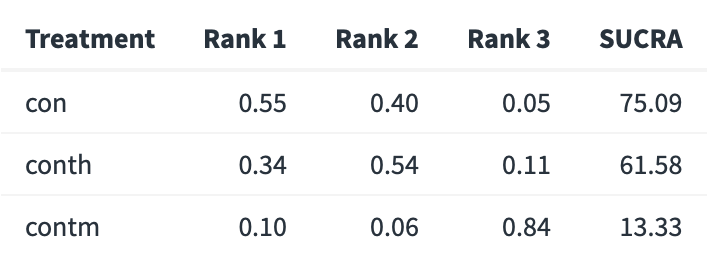

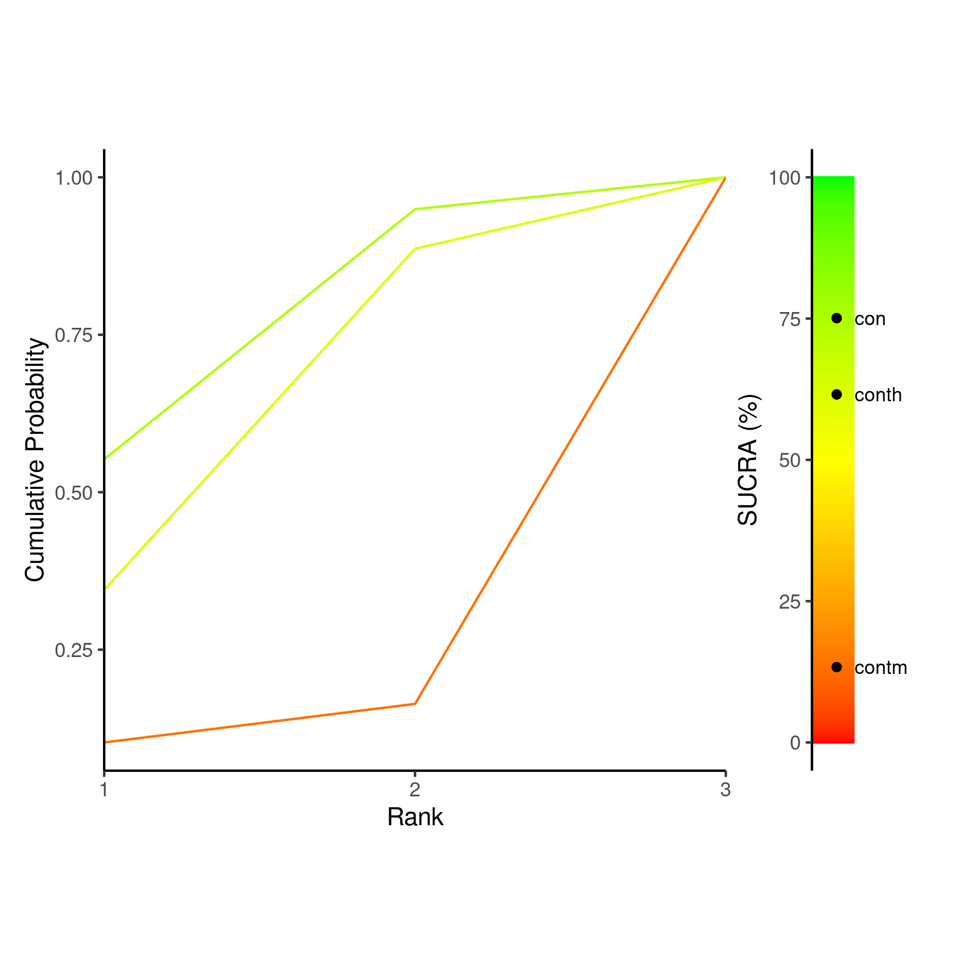


- Change in Serum Creatinine.


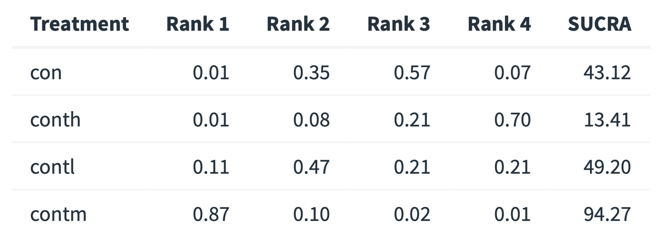

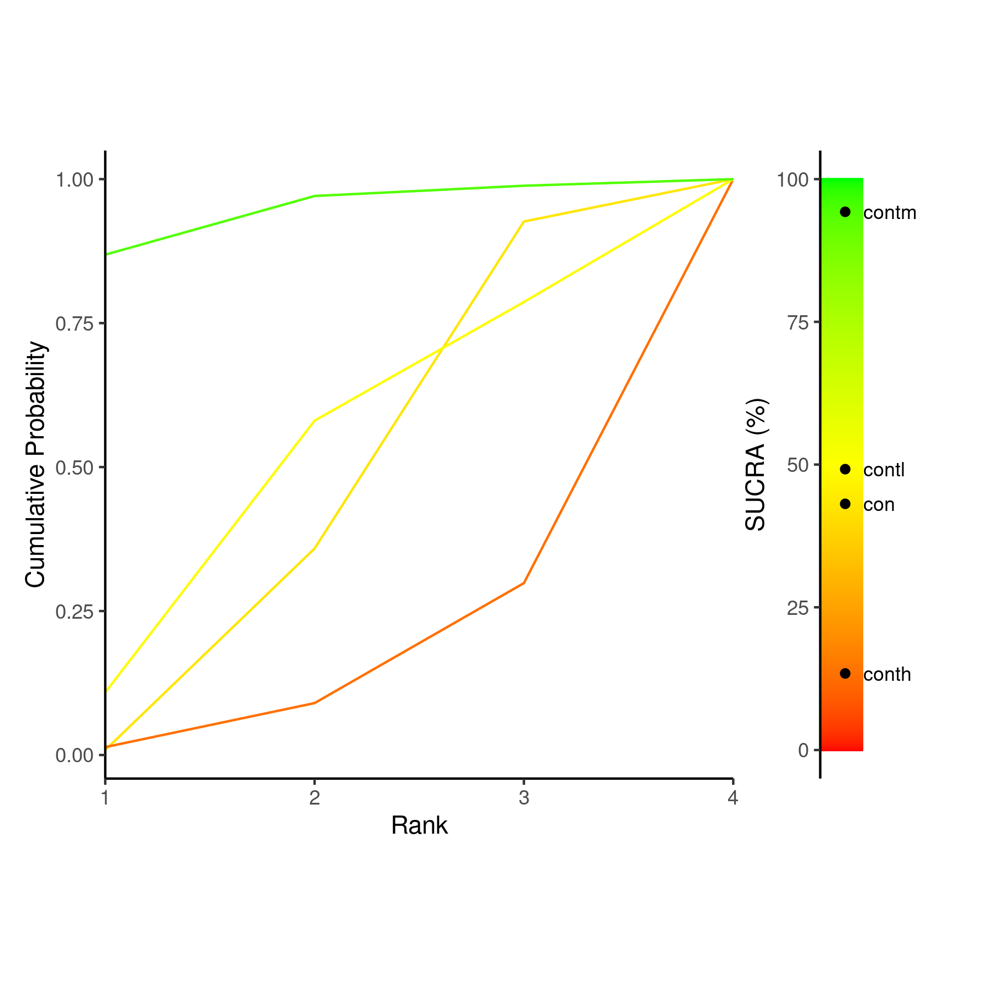


- Change in Serum Sodium.


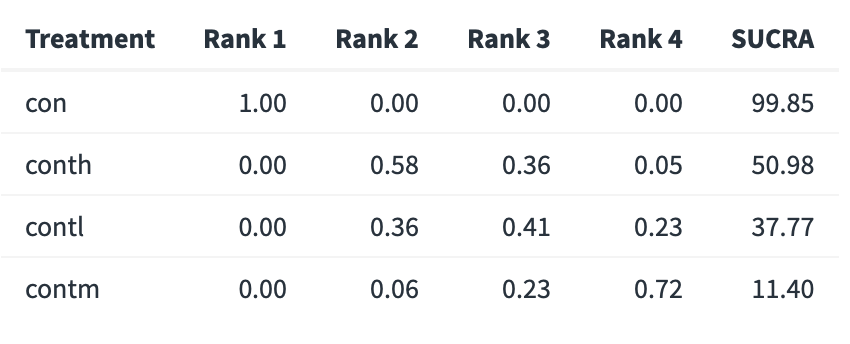

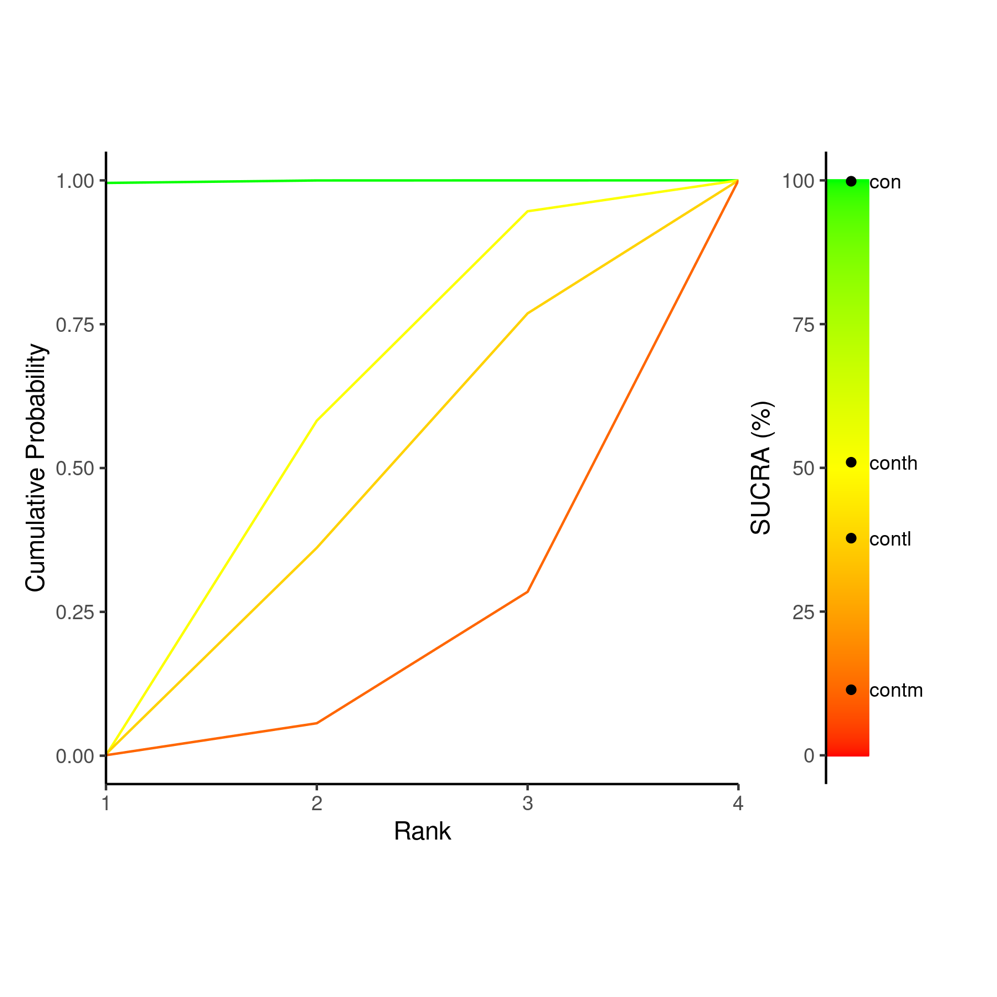


- Mortality.


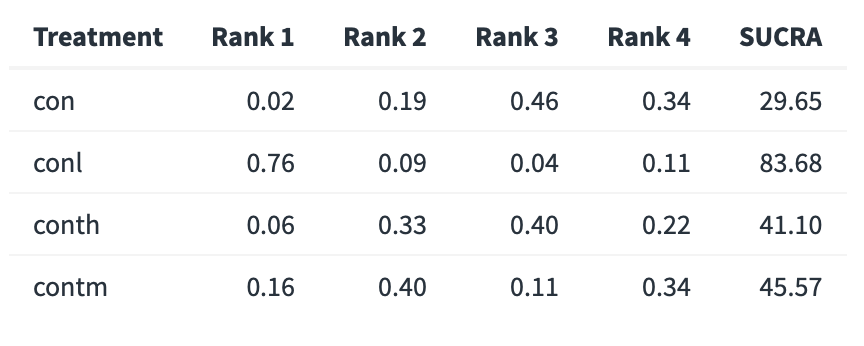

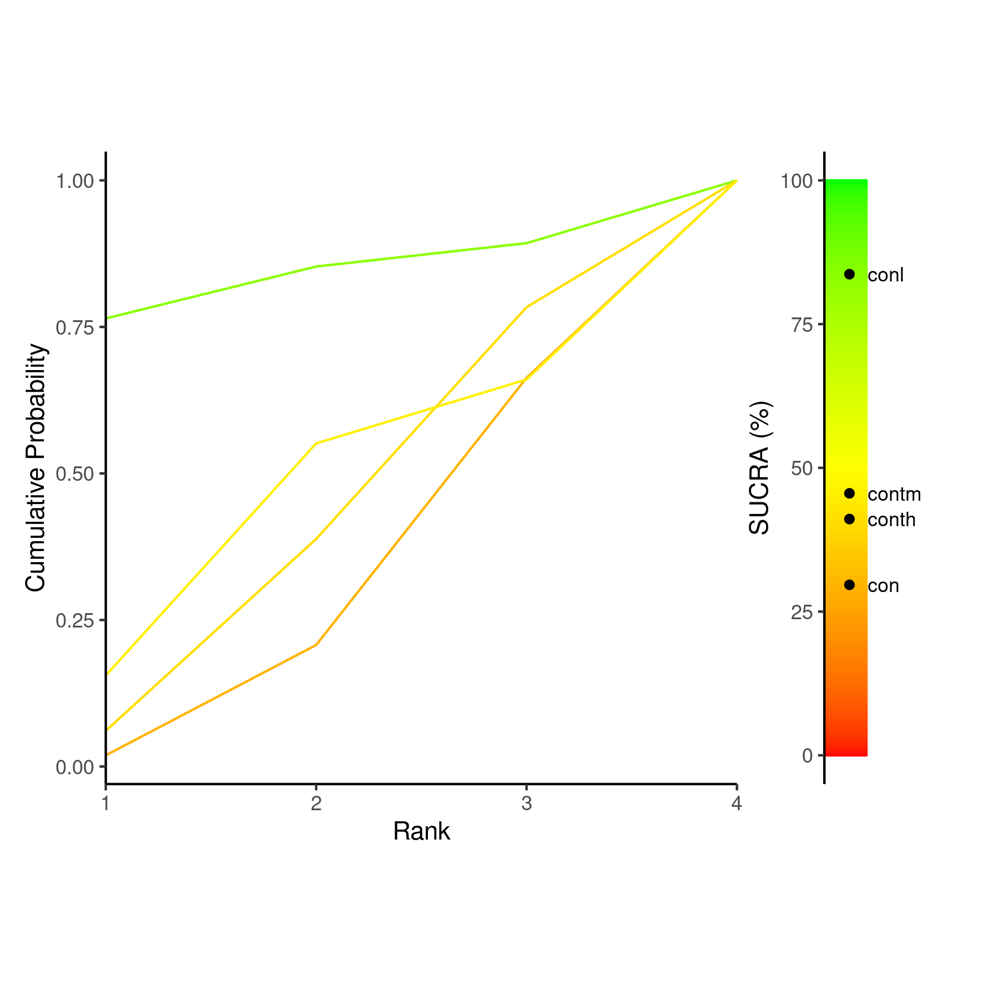

Supplement: Supplementary file 2 [file Datasheet1.zip › Data Sheet 1_v1/Supplementary 6.DOCX]
