# Supplementary material for: The role of tolvaptan add-on therapy in patients with acute heart failure: a systematic review and network meta-analysis
Source: Front Cardiovasc Med. 2024 May 30;11:1367442. doi: 10.3389/fcvm.2024.1367442 (PMC11169583; doi:10.3389/fcvm.2024.1367442)

## Supplementary 7.

**League Tables**. Pooled estimates for each dosage of the network meta-analysis.

- Dyspnea Relief within 24h.


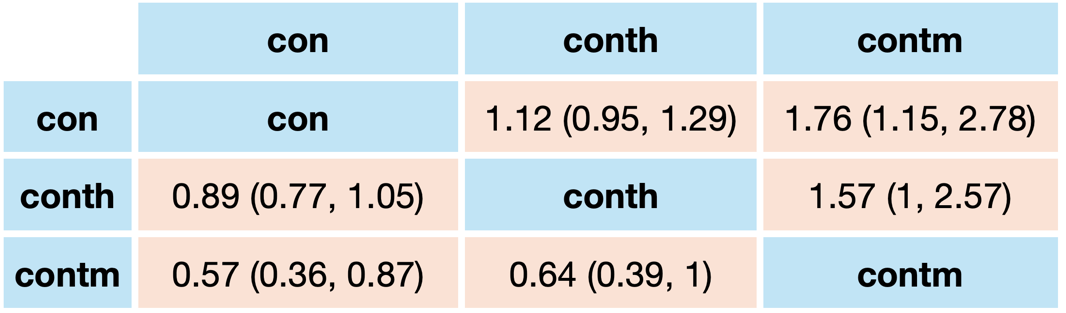


- Dyspnea Relief within 48h.


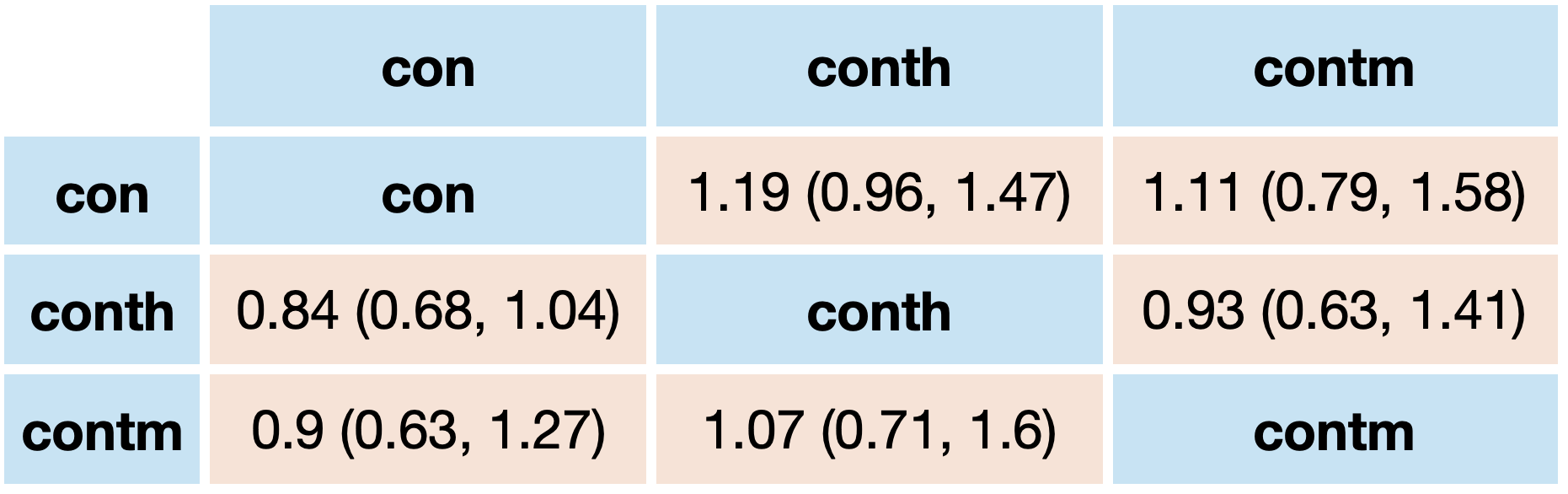


- Change in Weight up to 48h.


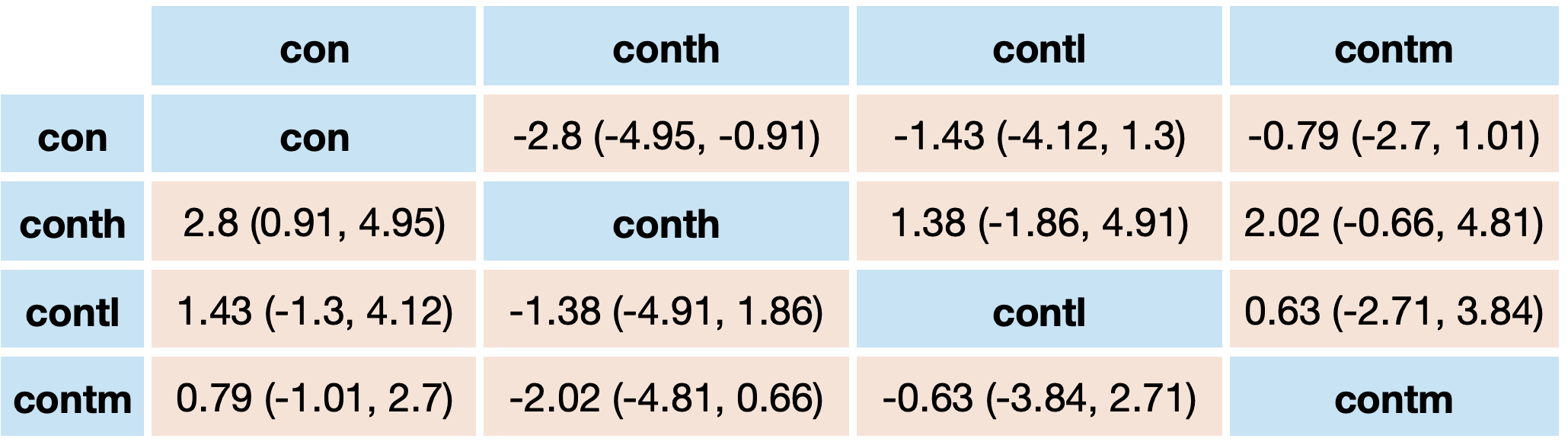


- Change in Weight up to 7 days.


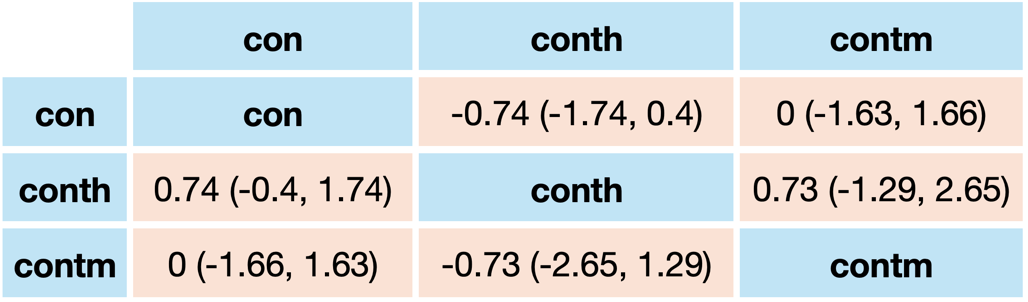


- Edema Reduction.


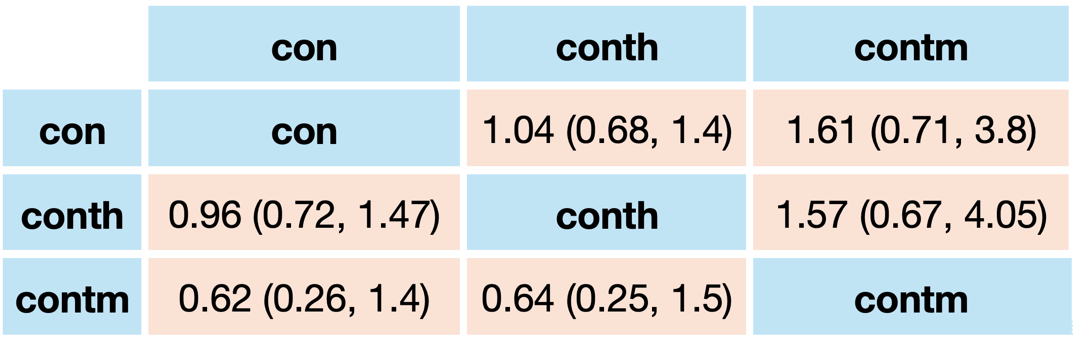


- Change in Serum Creatinine.


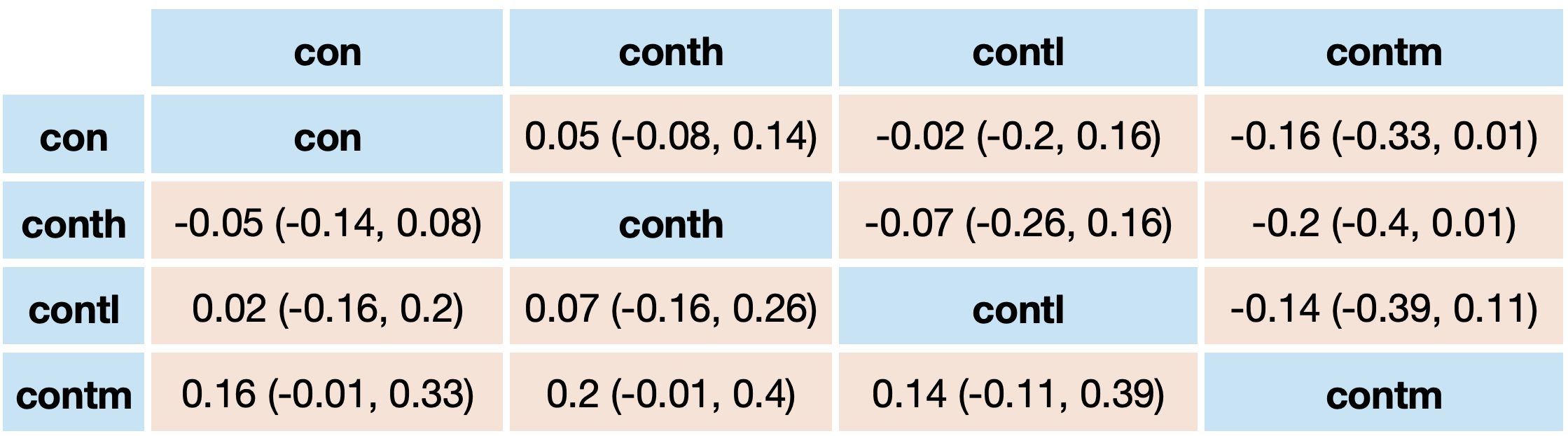


- Change in Serum Sodium.


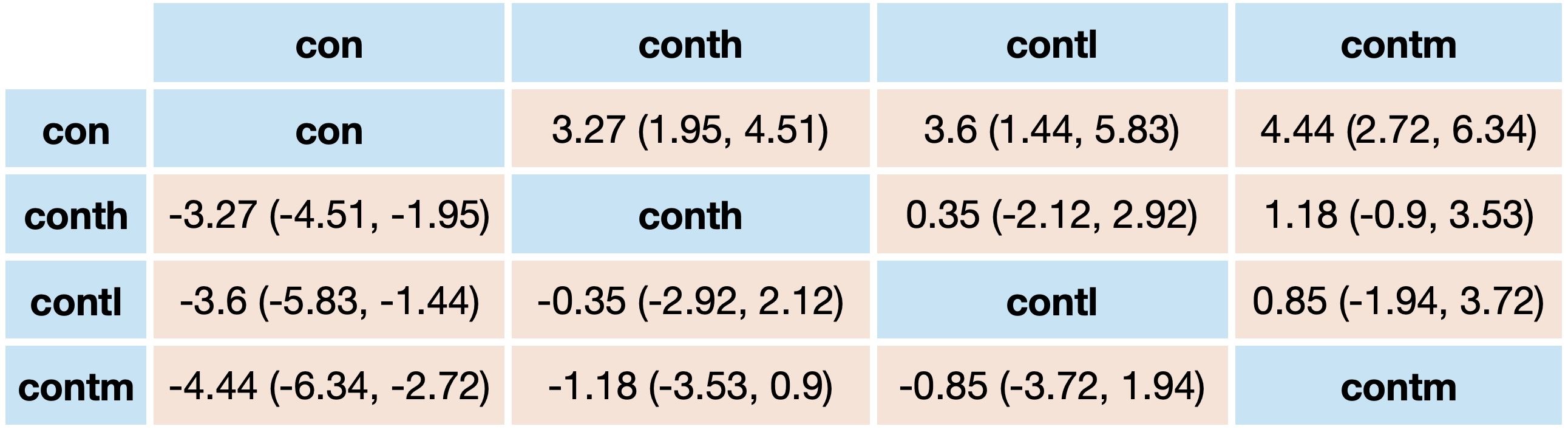


- Mortality.


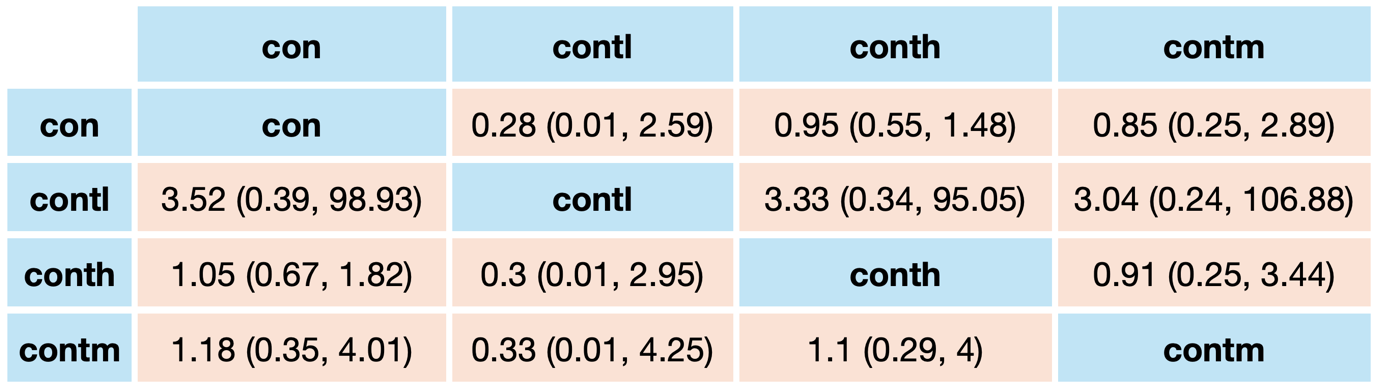

Supplement: Supplementary file 2 [file Datasheet1.zip › Data Sheet 1_v1/Supplementary 7.DOCX]
