# Supplementary material for: The role of tolvaptan add-on therapy in patients with acute heart failure: a systematic review and network meta-analysis
Source: Front Cardiovasc Med. 2024 May 30;11:1367442. doi: 10.3389/fcvm.2024.1367442 (PMC11169583; doi:10.3389/fcvm.2024.1367442)

## Supplementary 8.

**Network plots for outcomes in network meta-analysis of add-on tolvaptan dosage strategies for AHF**. con: conventional therapy; contl: conventional therapy + Tolvaptan low dose; contm: conventional therapy + tolvaptan intermediate dose; conth: conventional therapy + tolvaptan high dose.

Dyspnea Relief within 24h. Dyspnea Relief within 48h.


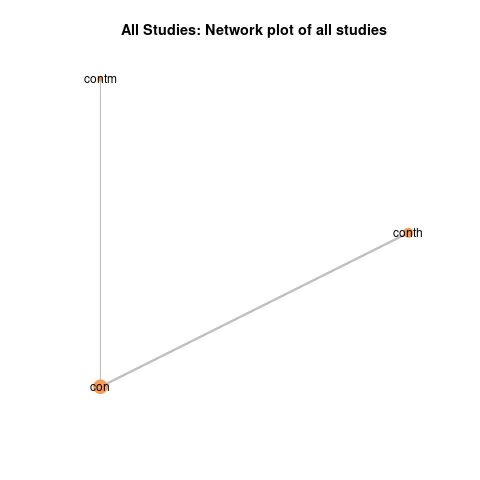

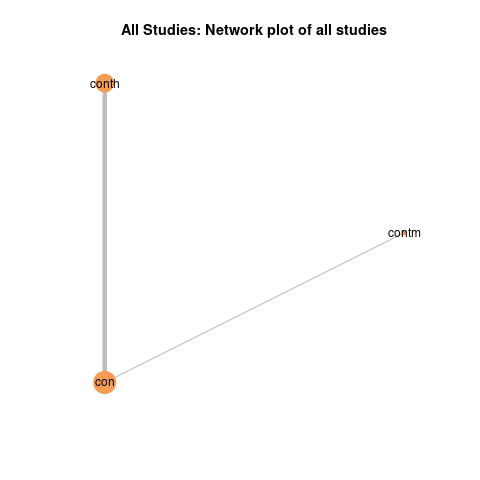


Change in Weight up to 48h. Change in Weight up to 7 days.


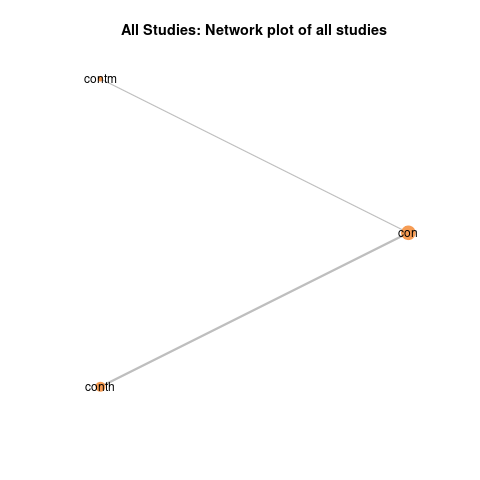

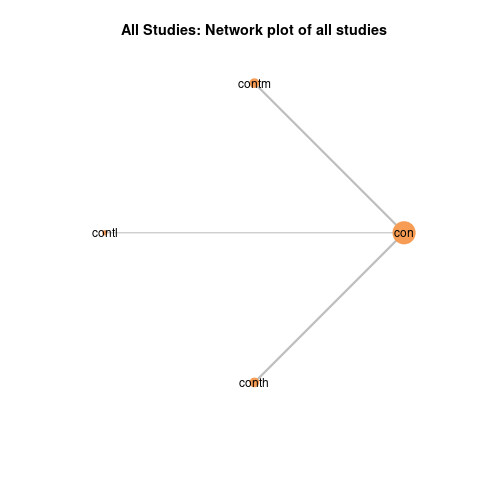


Edema Reduction. Change in Serum Creatinine.


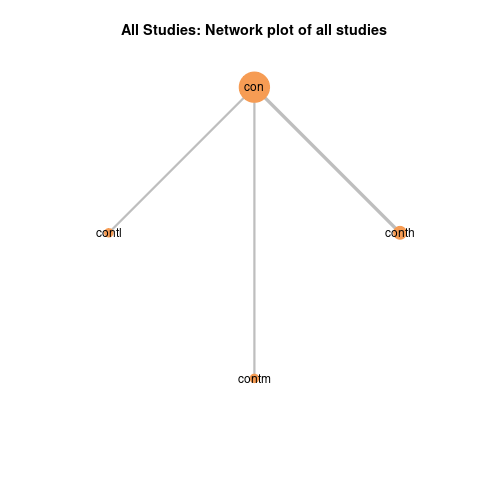

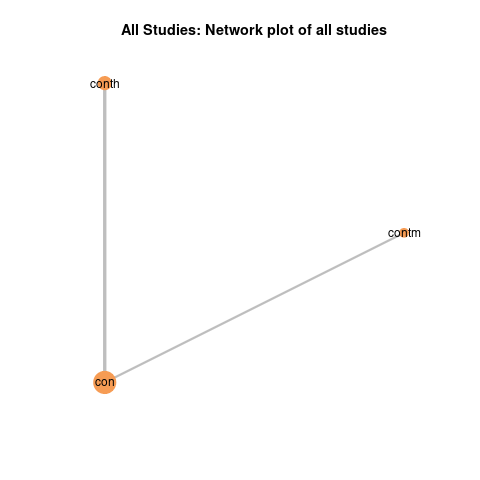


Change in Serum Sodium. Mortality.


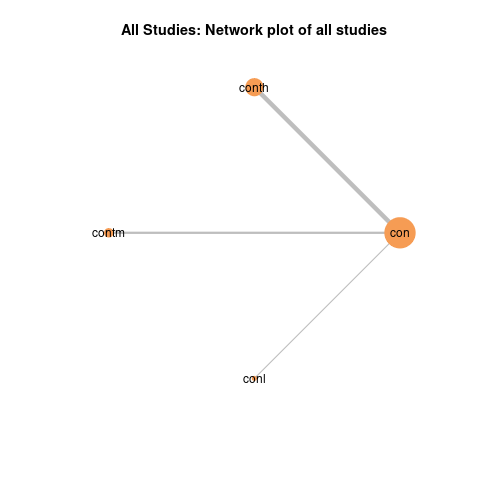

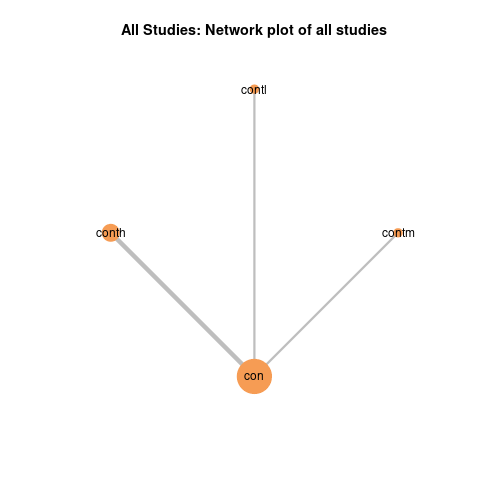

Supplement: Supplementary file 2 [file Datasheet1.zip › Data Sheet 1_v1/Supplementary 8.DOCX]
